# Cell-Free but potent: Antimicrobial potential of *Lactobacillus* supernatants against multidrug-resistant pathogens

**DOI:** 10.1371/journal.pone.0356730

**Published:** 2026-08-25

**Authors:** Sandra Pamela Cangui-Panchi, Diego Andrés Villavicencio-Pilacuan, Patricio Rojas-Silva, José F. Álvarez-Barreto, Andrés S. Lagos, José R. Mora, Eduardo Tejera, Jorge Reyes-Chacón, Daniel Garzon-Chavez, José M. Álvarez-Suarez, António Machado

**Affiliations:** 1 Laboratorio de Bacteriología, Instituto de Microbiología, Colegio de Ciencias Biológicas y Ambientales (COCIBA), Universidad San Francisco de Quito (USFQ), Quito, Ecuador; 2 Facultad de Ciencias Químicas, Universidad Central del Ecuador (UCE), Quito, Ecuador; 3 Laboratorio de Cultivo Celular, Instituto de Microbiología, Colegio de Ciencias Biológicas y Ambientales (COCIBA), Universidad San Francisco de Quito (USFQ), Quito, Ecuador; 4 Departamento de Ingeniería Química, Colegio de Ciencias e Ingenierías, Universidad San Francisco de Quito (USFQ), Quito, Ecuador; 5 Departamento de Ingeniería Química, Instituto de Simulación Computacional (ISC-USFQ), Universidad San Francisco de Quito (USFQ), Quito, Ecuador; 6 Facultad de Ingeniería y Ciencias Agropecuarias Aplicadas, Grupo de Bio-Quimioinformática, Universidad de Las Américas (UDLA), Quito, Ecuador; 7 Hospital General del Sur de Quito del Instituto Ecuatoriano de Seguridad Social (IESS), Quito, Ecuador; 8 Colegio de Ciencias de la Salud, Universidad San Francisco de Quito (USFQ), Quito, Ecuador; 9 Laboratorio de Investigación en Ingeniería en Alimentos (LabInAli), Departamento de Ingeniería en Alimentos, Colegio de Ciencias e Ingenierías, Universidad San Francisco de Quito (USFQ), Quito, Ecuador; 10 Laboratorio de Bioexploración, Colegio de Ciencias Biológicas y Ambientales, Universidad San Francisco de Quito (USFQ), Quito, Ecuador; 11 Laboratório de Biofilmes, Departamento de Biologia, Faculdade de Ciências e Tecnologia, Universidade dos Açores, Ponta Delgada, Portugal; 12 CIBIO, Centro de Investigação em Biodiversidade e Recursos Genéticos, InBIO Laboratório Associado, BIOPOLIS Programa em Genómica, Biodiversidade e Planeamento; Cátedra Unesco Terra no Mar: Biodiversidade e Sustentabilidade em Ilhas Atlânticas, Universidade dos Açores, Ponta Delgada, Portugal; Universidad Autonoma de Chihuahua, MEXICO

## Abstract

Antimicrobial resistance poses a major threat to public health and the food industry, driving the search for novel biologically derived antimicrobial alternatives. This study evaluated the antimicrobial activity of cell-free supernatants (CFS) obtained from 30 *Lactobacillus* strains against 15 bacterial and yeast pathogens, including multidrug-resistant isolates. A species-independent antimicrobial response was observed, with 10 CFS classified as effective, showing predominant MIC and MBC/MFC values of 20 mg/mL, in contrast to 5 ineffective CFS that had higher values despite some being of the same species. Effective CFS caused marked reductions in microbial viability, reaching 2–4 log (CFU/mL) decreases, and residual viable counts between 10^4^ and 10^5^ cells/cm^2^, as determined by fluorescence microscopy. Neutralization of CFS pH and enzymatic treatments resulted in a partial loss of antimicrobial activity, indicating that both acidic and non-acidic metabolites contribute to the observed effects. Cytotoxicity assays revealed no significant impact on HepG2 cell viability, whereas RAW 264.7 macrophages exhibited a reduction in MTT signal, which may reflect altered metabolic activity or increased cellular sensitivity rather than definitive cytotoxicity. Overall, *Lactobacillus*-derived CFS exhibit broad-spectrum antimicrobial activity associated with a diverse chemical composition, supporting their prioritization for further chemical characterization and translational studies.

## Introduction

The declining effectiveness of antibiotics and antifungals has led antimicrobial resistance (AMR) to emerge as one of the major threats to public health and the food industry, contributing to increased morbidity and mortality, a higher risk of infectious outbreaks, compromised food safety, and substantial economic losses [1–3]. According to the World Health Organization (WHO), 1.27 million deaths worldwide were directly attributable to AMR in 2019, and projections estimate that, in the absence of effective interventions, AMR could cause up to 28.03 million deaths annually by 2050 [4–6]. This alarming scenario has intensified the search for novel and complementary antimicrobial strategies, particularly those derived from biological sources with bactericidal and fungicidal potential [7–9].

Among biologically derived antimicrobials, postbiotics produced by *Lactobacillus* spp., including cell-free supernatants (CFS), have gained increasing attention due to their broad antimicrobial activity. These supernatants contain a complex mixture of bioactive compounds, such as organic acids, proteins, peptides, hydrogen peroxide, and bacteriocins, which together contribute to their bactericidal and fungicidal effects [10–12]. Importantly, CFS-based postbiotics do not rely on a single active component; instead, their antimicrobial activity arises from simultaneous and complementary mechanisms of action, which may reduce the likelihood of resistance development through microbial adaptation or evolution [13,14]. In this context, bacteriocins, as ribosomally synthetized peptides commonly found in *Lactobacillus* CFS, are of particular interest, as they target highly conserved and essential microbial structures potentially limiting resistance emergence [12,15–17].

Recent evidence has underscored the importance of CFS derived from *Lactobacillus* and their potential antimicrobial activity, which does not depend solely on acidification of the environment. Santarelli et al. demonstrated that the CFS from *Lactobacillus crispatus* M247 significantly reduced the viability of clinically relevant vaginal pathogens, including *Escherichia coli*, *Klebsiella pneumoniae*, *Staphylococcus aureus*, *Streptococcus agalactiae*, *Enterococcus faecalis*, and *Candida albicans*. Furthermore, they reported that the antimicrobial activity of the CFS was pH-dependent but was not affected by exposure to temperature or treatment with proteinase K, demonstrating that the antimicrobial effects of CFS are multifactorial [18]. Complementarily, recent evidence from a comparative genomic analysis of *L. crispatus* strains assessed the diversity of bacteriocin genes and their relationship to strain-specific adaptation, microbial competitiveness, and probiotic potential. This perspective is relevant because the presence, conservation, or variability of bacteriocin-associated genes may help explain functional differences among isolates, even within the same species [19].

An additional advantage of CFS lies in their classification as postbiotics, defined as non-viable microbial products derived from beneficial microorganisms [20]. This non-viable nature enhances safety, stability, and shelf life, facilitating their application on living tissues and inert surfaces, as well as enabling diverse routes of administration and easier transport and storage when compared with live probiotics [21,22].

Despite growing evidence supporting the antimicrobial activity of *Lactobacillus* CFS, significant knowledge gaps remain. Although there is recent evidence regarding the antimicrobial activity of CFS derived from lactic acid bacteria against clinically relevant and multidrug-resistant pathogens, most studies focus on a limited number of CFS tested against a small subset of microorganisms, hindering the identification of broad-spectrum antimicrobial profiles [23–29]. Therefore, the objective of this study was to evaluate, from a phenotypic and comparative perspective, the antimicrobial potential of CFS derived from 30 *Lactobacillus* strains isolated from vaginal microbiota against 15 bacterial and yeast pathogens, including multidrug-resistant isolates. In addition, the chemical composition of the CFS was characterized exploratorily by quantifying carboxylic acids, free amino acids, and total proteins, estimating the contribution of acidic and non-acidic components through pH neutralization and enzymatic treatments, and evaluating the cytotoxic response of selected CFS in HepG2 and RAW 264.7 cell lines.

## Results and discussion

### Identification and characterization of *Lactobacillus* and pathogenic strains

Thirty *Lactobacillus* strains were assigned to nine taxonomic groups; *Lactobacillus gasseri* was the most prevalent species (n = 11), followed by *Lactobacillus crispatus* (n = 5). Other identified taxa included *Lactiplantibacillus plantarum* (n = 2), *Lactobacillus acidophilus* (n = 2), *Lactobacillus iners* (n = 1), *Limosilactobacillus reuteri* (n = 1), *Limosilactobacillus fermentum* (n = 1), *Lactobacillus jensenii* (n = 1), and *Lactobacillus* spp. (n = 6) (S1 Table).

The identification of the 15 pathogenic microorganisms was initially realized via PCR (S2 Table) and then corroborated at the genus and species levels using MALDI-TOF-MS, with all strains yielding score values > 2.00, indicating high confidence and consistency of identification (S3 Table). The collection comprised both reference and multidrug-resistant (MDR) strains. Gram-negative bacteria included *Escherichia coli* (n = 2), *Klebsiella pneumoniae* (n = 3), and *Pseudomonas aeruginosa* (n = 2). Gram-positive bacteria comprised *Staphylococcus aureus* (n = 2), *Enterococcus faecalis* (n = 2), and *Enterococcus faecium* (n = 1). In addition, three yeast strains were included, corresponding to *Candida albicans* (n = 1) and *Candida tropicalis* (n = 2). This panel of microorganisms enabled the standardized evaluation of the antimicrobial activity of CFS against clinically relevant bacterial and fungal pathogens. MALDI-TOF-MS is widely used in clinical microbiology for microbial identification at the proteomic level, offering high accuracy at the species level while emphasizing speed, performance, and cost-effectiveness [30,31]. Nevertheless, limitations related to database coverage and spectrum quality may result in reduced resolution at the strain level [32].

### Variability of the chemical composition in *Lactobacillus* CFS

**Fig 1:**
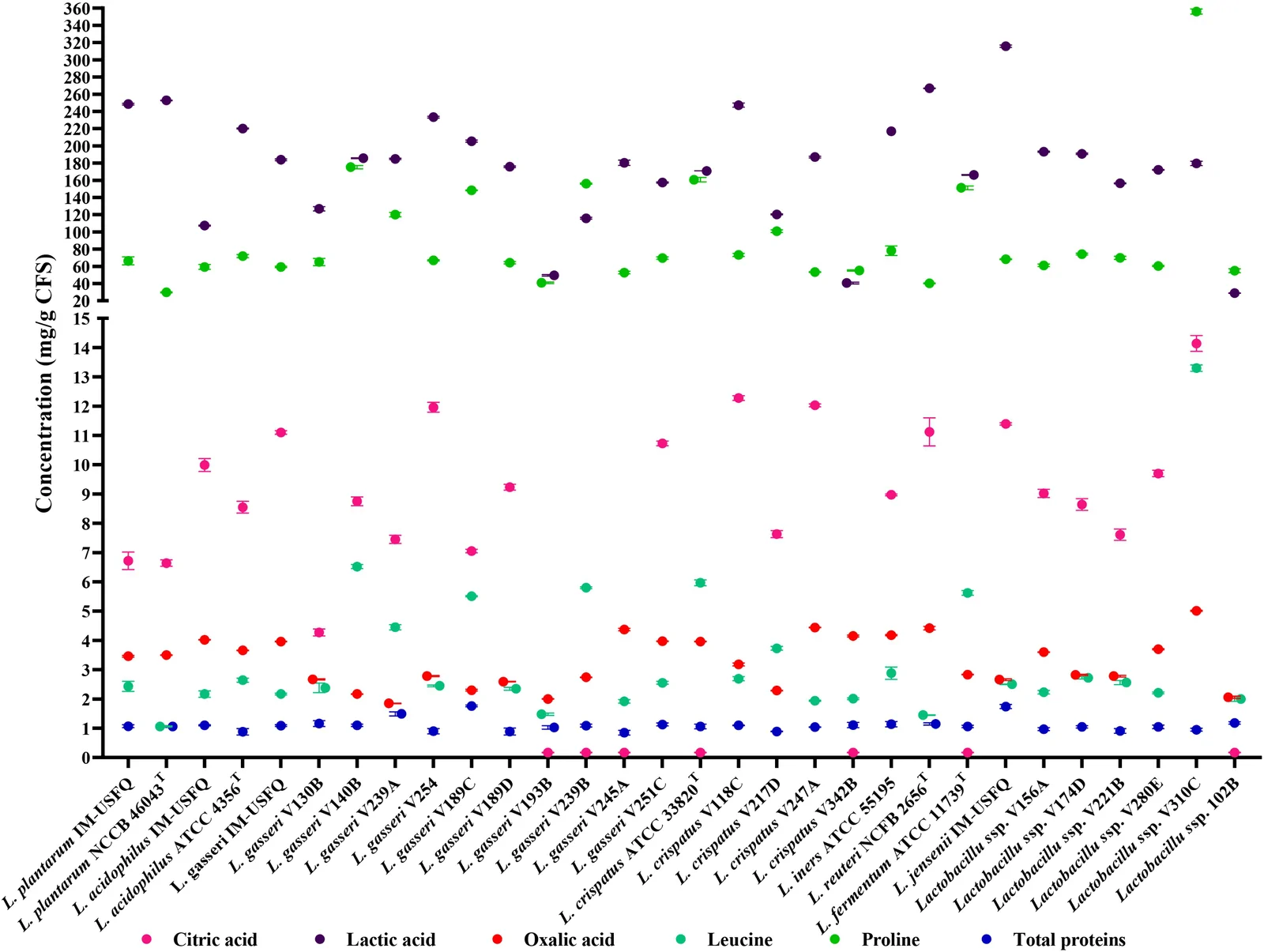
Metabolite concentrations in CFS from *Lactobacillus* strains. Color-coded dots represent values for each metabolite. The bar represents median values with standard deviation (SD).

Prior to evaluating antimicrobial activity, the chemical composition of CFS obtained from 30 *Lactobacillus* strains was characterized (Fig 1). Detailed quantitative data for each metabolite are provided in S4 Table.

Overall, the HPLC-based quantification of carboxylic acids showed substantial inter-strain variability, with a metabolic profile clearly dominated by lactic acid, whose concentrations ranged from 28.94 to 318.85 mg/g CFS. In contrast, citric acid and oxalic acid were detected at markedly lower levels, ranging from 0.17 to 14.14 mg/g CFS and from 1.85 to 5.01 mg/g CFS, respectively. Malic acid was not quantified, as its signal remained below the quantification limit in all samples (LOQ = 5.972 ppm).

The predominance of lactic acid is consistent with the physiological characteristics of lactic acid bacteria, for which lactic acid production represents a central metabolic outcome. However, the amount produced varied considerably among strains, reflecting differences in substrate availability, growing conditions, and genetic determinants [33]. These findings agree with the study by Hu et al., who reported the highest lactic acid concentrations in CFS, particularly for *L. plantarum* S11 (26.4 g/L, equivalent to 26.4 mg/g assuming *p* = 1 g/mL), together with very low malic acid levels (<0.4 g/L, equivalent to <0.4 mg/g assuming *p* = 1 g/mL) [34]. Similarly, Rahman et al. reported lactic acid concentrations of 6.01 and 5.81 mg/mL (equivalent to 6.01 and 5.81 mg/g assuming *p* = 1 g/mL) in freeze-dried CFS from *L. acidophilus* ATCC 4356 and *L. plantarum* NBRC 3070, respectively [35]. The comparatively low levels of oxalic acid observed in the present study are consistent with those reported by Bolívar-Jacobo et al., who described oxalic acid concentrations ranging from 0.013 to 0.102 mM (equivalent to 0.001 and 0.01 mg/g assuming *p* = 1 g/mL), alongside higher levels of citric acid (1.051–6.115 mM, equivalent to 0.22 and 1.28 mg/g assuming *p* = 1 g/mL) and lactic acid (15.710–927.51 mM, equivalent to 1.41 and 85.55 mg/g assuming *p* = 1 g/mL) in *L. acidophilus* and *L. helveticus* strains [36]. It should be noted that this analysis involved a targeted quantification of four carboxylic acids and not a comprehensive metabolomic characterization of the CFS.

In addition to organic acids, colorimetric assays revealed concentrations of free amino acids, with leucine ranging from 1.06 to 13.30 mg/g CFS and proline from 29.74 to 356.03 mg/g CFS, while total protein content varied between 0.85 and 1.76 mg/g CFS. Taken together, these results demonstrate pronounced differences in the chemical composition of CFS among strains, largely independent of species-level classification. Such variability likely reflects differences in the activity and regulation of the proteolytic systems of individual lactic acid bacteria [37]. Rozhkova et al. reported leucine + isoleucine concentrations of 0.29 and 0.46 mg/100 mL of CFS from *L. helveticus* H9 and *L. paracasei* ABK, respectively, values comparable to those observed in the present study; however, reported proline levels (0.69–0.90 mg/100 mL) were substantially lower than the wide range detected here [38]. The relatively low protein content observed may be attributed to the dominant contribution of organic acids and the strain-specific proteolytic activity of these bacteria [39]. Overall, the protein concentrations obtained are consistent with those reported by Rahman et al., who described values ranging from 0.84 to 0.98 mg/mL of CFS across five *Lactobacillus* species [35].

### Comparative antimicrobial profiles of CFS demonstrate broad-spectrum activity

In the initial screening, the antimicrobial activity of CFS obtained from 30 *Lactobacillus* strains was evaluated against a panel of 15 pathogenic microorganisms, including multidrug-resistant strains, by determining the MIC using microdilution assays. In this collection of vaginal *Lactobacillus* strains, MIC values did not cluster consistently by species but instead showed marked strain-dependent variability. Notably, ten CFS derived from different *Lactobacillus* species displayed broad-spectrum antimicrobial activity, inhibiting the growth of all tested pathogens at concentrations ranging from 10 to 40 mg/mL (S1 Fig).

The observed variability in MIC values is consistent with previous reports demonstrating strain-dependent antimicrobial activity among *Lactobacillus* spp. For instance, Arena et al. described heterogeneous inhibition rates (10–90%) among 17 *L. plantarum* strains tested against *E. coli* O157:H7, *L. monocytogenes*, and *S.* Enteritidis [40]. Similarly, Scillato et al. reported differential inhibitory effects of CFS from *L. gasseri*, *L. fermentum*, and *L. crispatus* against clinically relevant pathogens, highlighting one *L. fermentum* strain with pronounced broad-spectrum antimicrobial activity [24].

**Fig 2:**
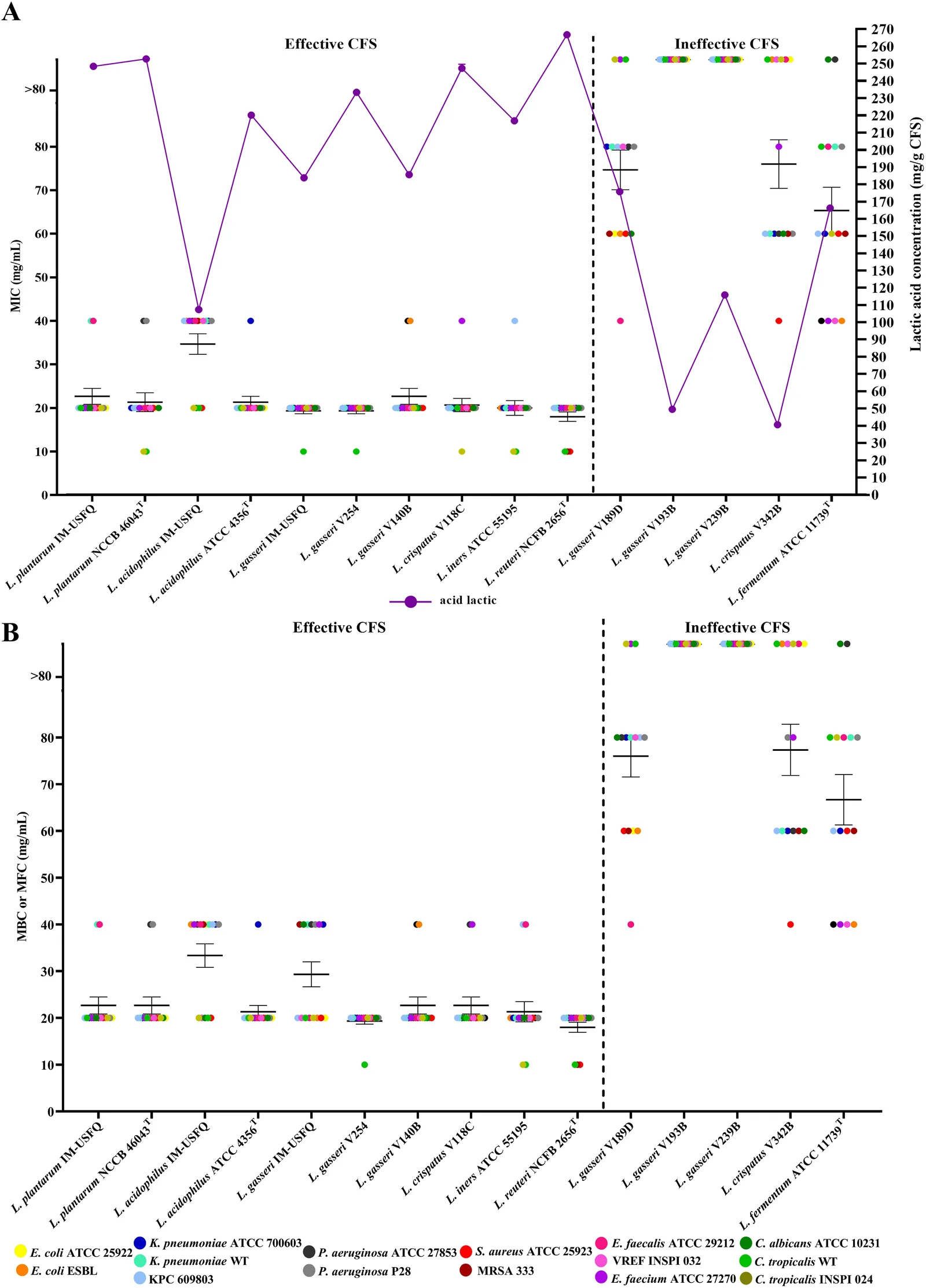
Comparison of MIC and MBC or MFC values between effective and ineffective CFS. (A) MIC values with lactic acid concentration and (B) MBC or MFC values of ten effective and five ineffective CFS against the pathogen panel. Each colored dot represents an individual pathogen. Purple lines and dots represent lactic acid concentration. Bars indicate median values with standard error of the mean (SEM).

Based on the initial screening, ten CFS with broad-spectrum antimicrobial activity and lower MIC and MBC or MFC were selected and classified as effective CFS. This group included CFS from strains belonging to the species *L. plantarum*, *L. acidophilus*, *L. gasseri*, *L. crispatus*, *L. iners*, and *L. reuteri*, and in most cases, the MIC and MBC or MFC values were 20 mg/mL against the fifteen pathogens. In contrast, five CFS exhibiting reduced antimicrobial activity were classified as ineffective CFS, showing MIC and MBC or MFC values ≥ 40 mg/mL. These ineffective CFS originated from strains of *L. gasseri*, *L. crispatus*, and *L. fermentum* (Fig 2). As lactic acid concentrations were the highest, they were compared among the selected CFS, determining that the range among effective CFS was 107.42 to 266.81 mg/g CFS, while the range among ineffective CFS was 40.61 to 175.78 mg/g CFS. Although the upper limit of lactic acid concentration in effective CFS is higher than in ineffective CFS, at intermediate concentrations there may or may not be antimicrobial potential, which is evident in the effective CFS *L. acidophilus* IM-USFQ that had a lactic acid concentration of 107.42 mg/g CFS, close to the 115.84 mg/g CFS of the ineffective *L. gasseri* V239B, which could indicate that there are other metabolites that could increase or inhibit antimicrobial activity.

Antibiotics and antifungals presented MIC and MBC/MFC values ranging from 0.000625 to 0.5 mg/mL in reference strains and values up to 10 mg/mL in resistant strains. It is important to emphasize that effective CFS had a broader antimicrobial spectrum compared to antimicrobials in resistant strains. Statistical analysis using the paired Wilcoxon signed-rank test showed that both MIC and MBC or MFC values obtained with effective CFS treatments were significantly lower than those observed for ineffective CFS across all pathogens (MIC: V = 0, *p* = 0.0005; MBC/MFC: V = 0, *p* = 0.0003, respectively). Detailed inhibition percentages at MIC and MBC or MFC concentrations, as well as the corresponding statistical significance relative to untreated controls, are provided in S5 and S6 Tables.

**Fig 3:**
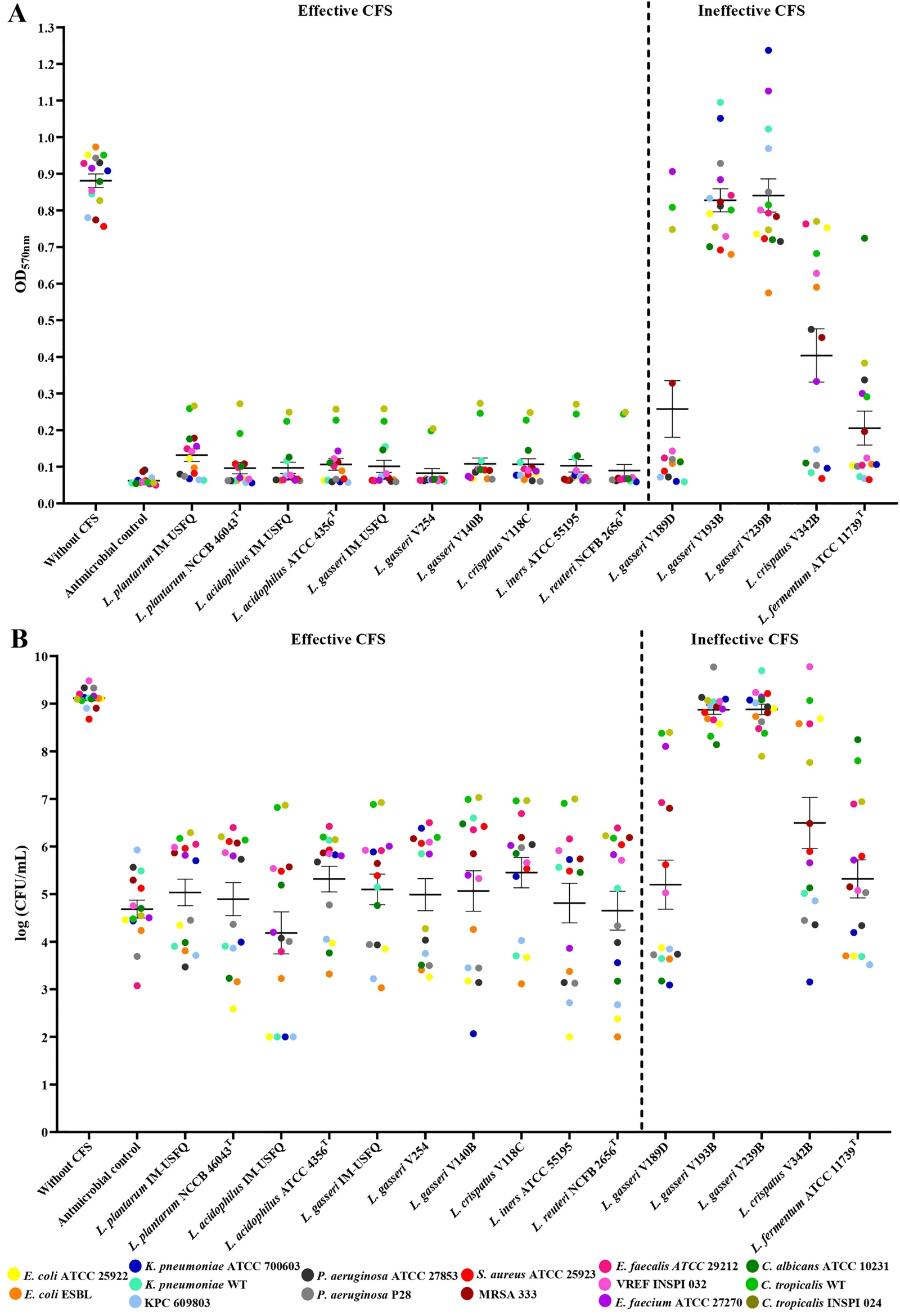
Effects of CFS on microbial growth and viability. (A) OD_570nm_ values and (B) log (CFU/mL) values for the ten effective and five ineffective CFS against the pathogen panel. Antimicrobial control tested with ciprofloxacin, ampicillin, and fluconazole according to the pathogen. Colored dots represent individual pathogens. Bars indicate median values with standard error of the mean (SEM).

Microbial inhibition assays based on optical density (OD_570nm_) measurements (Fig 3A) and viable cell counts expressed as log (CFU/mL) (Fig 3B) demonstrated that treatments with effective CFS resulted in significantly lower values than those observed with ineffective CFS across all tested pathogens. According to the paired Wilcoxon signed-rank test, these differences were statistically significant for both OD₅₇₀ nm values (V = 1, *p* = 0.0009) and log (CFU/mL) values (V = 11, *p* = 0.0059). Mean OD₅₇₀ nm values, log (CFU/mL) data, and corresponding statistical significance relative to the untreated control are detailed in S7 and S8 Tables.

Notably, reductions in viable cell counts were not always proportional to decreases in OD_570nm_ values, indicating that growth inhibition does not necessarily correspond to bactericidal or fungicidal activity. This discrepancy can be explained by differences in the rate of antimicrobial action or the physiological state of each pathogen. However, no time-kill assays were conducted to corroborate these results. Nevertheless, effective CFS produced an average reduction of approximately 4 log (CFU/mL) relative to the control without CFS, representing a biologically meaningful antimicrobial effect with consistent statistical significance.

For Gram-negative bacteria, including *E. coli* ATCC 25922, *E. coli* ESBL, *K. pneumoniae* ATCC 700603, *K. pneumoniae* WT, KPC 609803, *P. aeruginosa* ATCC 27853, and *P. aeruginosa* P28, viable counts generally decreased to below 4.5 log (CFU/mL) following treatment with effective CFS. Among these, the CFS from *L. acidophilus* IM-USFQ showed the most pronounced effect, reducing viable counts to as low as 2 log (CFU/mL) against four pathogens, consistent with a bactericidal effect defined as a ≥ 3 log reduction [41,42]. Comparable reductions were also observed with CFS from *L. gasseri* V140B, *L. iners* ATCC 55195, and *L. reuteri* NCFB 2656^T^.

In contrast, fungal pathogens, including *C. albicans* ATCC 10231, *C. tropicalis* WT and *C. tropicalis* INSPI 024, exhibited more limited susceptibility, with viable counts typically ranging between 4 and 7 log (CFU/mL) after treatment, suggesting predominantly fungistatic activity. A similar trend was observed for Gram-positive bacteria, including *S. aureus* ATCC 25923, MRSA 333, *E. faecalis* ATCC 29212, VREF INSPI 032, and *E. faecium* ATCC 27270, which generally showed final counts between 4 and 6 log (CFU/mL).

These findings are consistent with previous studies reporting strain-dependent and pathogen-specific antimicrobial effects of *Lactobacillus* CFS. Scillato et al. described broad-spectrum activity of CFS from *L. gasseri* 1A-TV, *L. fermentum* 18A-TV, and *L. crispatus* 35A-TV, with bacterial reductions ranging from 1 to 4 log (CFU/mL) for *E. coli*, *S. agalactiae*, *S. aureus*, *K. pneumoniae*, *P. mirabilis*, and *P. vulgaris;* while *Candida* and *Enterococcus* species maintained higher viable counts (5 and 8 log CFU/mL) [24]. Similarly, Kim et al. reported reductions to 8 and 5 log (CFU/mL) for *E. coli* and *S. aureus*, respectively, following treatment with CFS from *L. plantarum* NIBR97 [16]. El-Mokhtar et al. observed decreases to 4 and 5 log (CFU/mL) for *K. pneumoniae* and *P. aeruginosa*, respectively, treated with *L. acidophilus* CFS [43] while Ołdak et al. reported reductions to 1 and 6 log (CFU/mL) for *L. monocytogenes* and *S.* Enteritidis, respectively, using CFS from two strains of *L. plantarum* [44].

### Fluorescence microscopic visualization confirms CFS-induced pathogen growth inhibition

**Fig 4:**
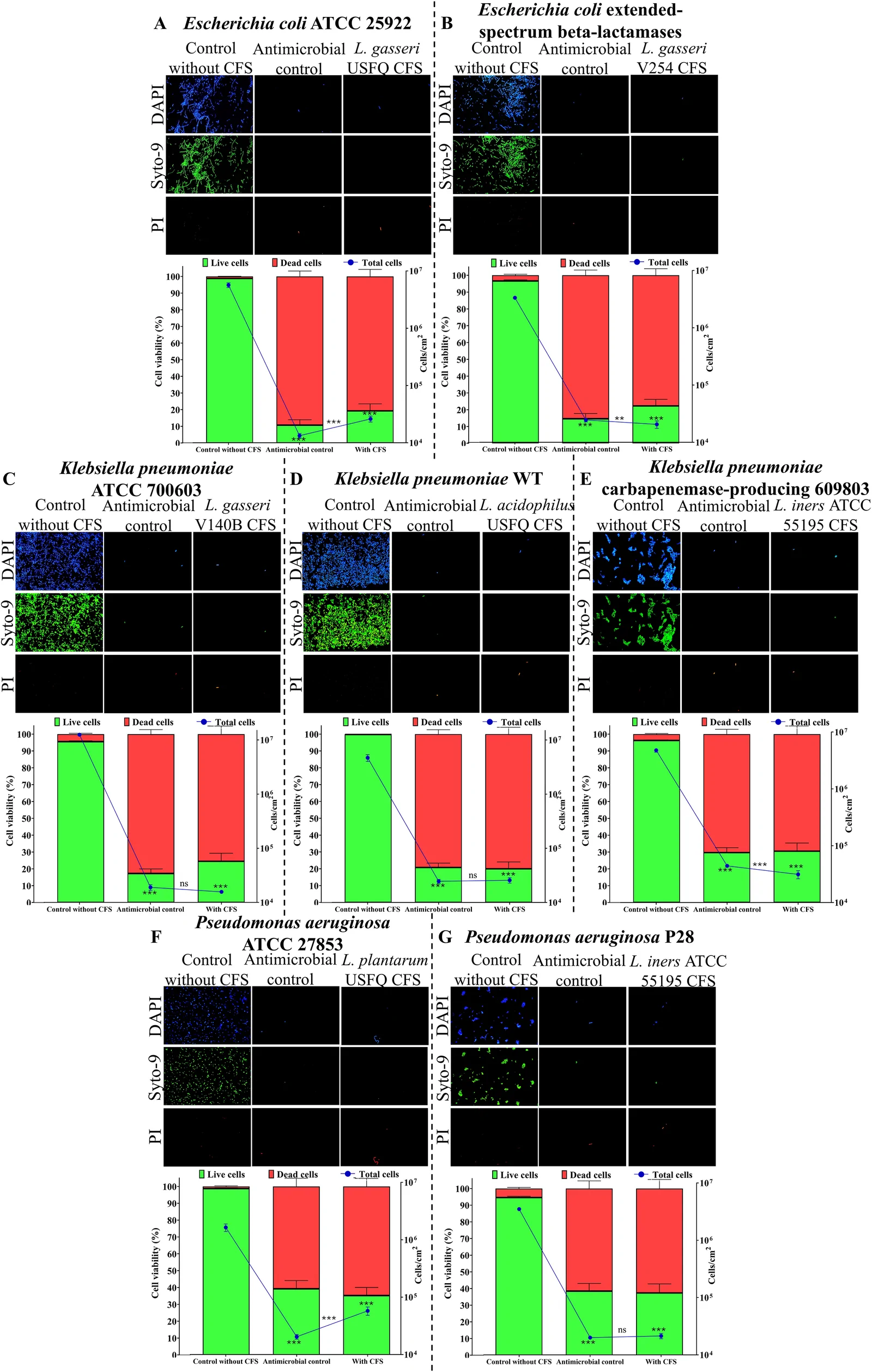
Microscopic visualization and cell viability of Gram-negative pathogens treated with CFS. (A, B) *E. coli* strains; (C-E) *K. pneumoniae* strains; (F, G) *P. aeruginosa* strains. DAPI stains total cellular DNA (blue), SYTO^TM^ 9 labels live cells (green), and propidium iodide (PI) marks dead cells (red). Bar graphs represent the percentage of live and dead cells, together with total cell density (cells/cm^2^) plotted on a logarithmic scale. Statistical differences were evaluated using the Wilcoxon test comparing untreated samples with antimicrobial controls and CFS treatments, as well as CFS versus antimicrobial controls (not significant, ns; *p* ≤ 0.05, *; *p* ≤ 0.01, **; *p* ≤ 0.001, ***). Bars represent mean values with standard error of the mean (SEM).

Fluorescence microscopy analyses were conducted using one effective CFS per pathogen to further assess its impact on cell viability. Among Gram-negative bacteria, treatment with CFS resulted in a pronounced reduction in viable cells. Cell viability decreased to approximately 20% in *E. coli* ATCC 25922 and *E. coli* ESBL (Fig 4A and 4B). In *K. pneumoniae* ATCC 700603, *K. pneumoniae* WT, and *K. pneumoniae* carbapenemase-producing 609803 viability values were reduced to approximately 25% (Fig 4C–4E). A comparatively smaller, yet still significant, reduction was observed for *P. aeruginosa* ATCC 27853 and *P. aeruginosa* P28, with viable cells representing approximately 35% of the population following CFS treatment (Fig 4F and 4G).

Consistent with these observations, total cell counts following CFS exposure ranged from 10^4^ to 10^5^ cells/cm^2^ across Gram-negative species/strains. A statistically significant reduction in cell density was observed for *E. coli* ESBL and KPC 609803 treated with CFS when compared to the antimicrobial control, indicating enhanced antimicrobial efficacy for these strains.

These findings align with previous reports demonstrating reduced pathogen viability following exposure to *Lactobacillus*-derived CFS using fluorescence-based approaches. For example, Aditya et al. reported a marked increase in dead (red-stained) cells in Shiga toxin-producing enterohemorrhagic *E. coli* O157:H7 treated with *L. casei* CFS [45]. Similarly, Nair et al. demonstrated a strong bactericidal effect against uropathogenic *E. coli* UTI89 using a combination of *Lactobacillus* CFS and tryptamine, as evidenced by live/dead fluorescence staining [46]. Moreover, confocal microscopy analyses have highlighted the antibiofilm activity of *Lactobacillus* CFS and its fractions, showing extensive cell death in *P. aeruginosa* strains isolated from chronic infections [47].

**Fig 5:**
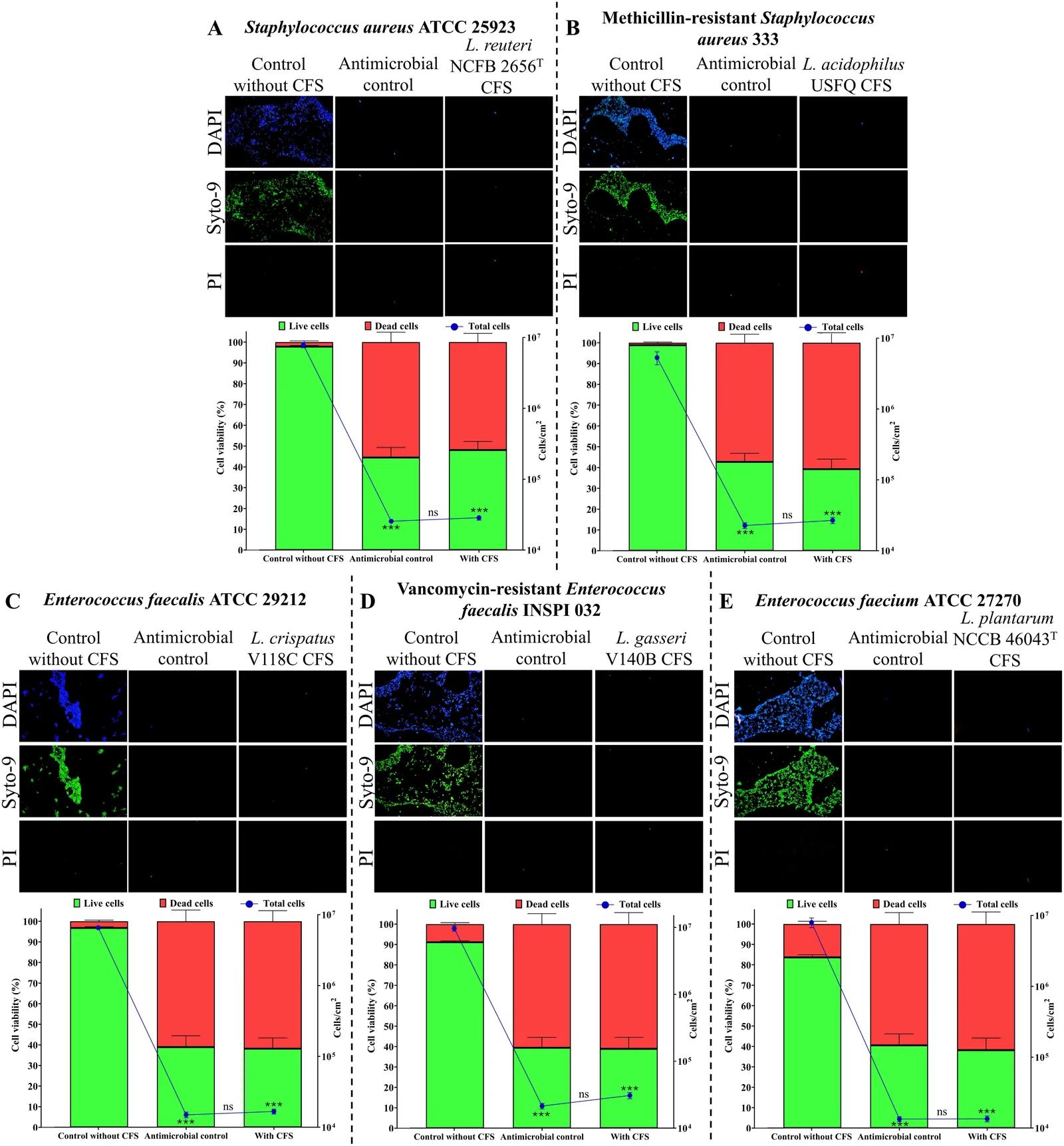
Microscopic visualization and cell viability of Gram-positive pathogens treated with CFS. (A, B) *S. aureus* strains; (C, D) *E. faecalis* strains; (E) *E. faecium* strain. DAPI stains total cellular DNA (blue), SYTO^TM^ 9 labels live cells (green), and propidium iodide (PI) marks dead cells (red). Bar graphs represent the percentage of live and dead cells, together with total cell density (cells/cm^2^) plotted on a logarithmic scale. Statistical differences were evaluated using the Wilcoxon test comparing untreated samples with antimicrobial controls and CFS treatments, as well as CFS versus antimicrobial controls (not significant, ns; *p* ≤ 0.05, *; *p* ≤ 0.01, **; *p* ≤ 0.001, ***). Bars represent mean values with standard error of the mean (SEM).

Among Gram-positive bacteria, fluorescence microscopy revealed a marked reduction in cell viability following treatment with CFS. The proportion of live cells decreased to approximately 48% in *S. aureus* ATCC 25923 (Fig 5A). In contrast, MRSA 333, *E. faecalis* ATCC 29212, VREF INSPI 032, and *E. faecium* ATCC 27270 exhibited lower viability levels, with live cells representing approximately 38% of the population after CFS treatment (Fig 5B–5E). Consistent with the observations made for Gram-negative pathogens, total cell densities following CFS exposure ranged from 10^4^ to 10^5^ cells/cm^2^, indicating that the reduction in viability was primarily associated with increased cell death rather than complete cell removal. These findings agree with previous microscopy-based studies reporting enhanced cell damage and death in Gram-positive pathogens exposed to *Lactobacillus*-derived CFS. Jurado et al. reported a higher proportion of dead or damaged cells in *S. aureus* biofilms treated with CFS from *L. fermentum* I7, *L. reuteri* 7SNG3–30, and *L. salivarius* 22SNG3–30 as visualized by confocal laser scanning microscopy [48]. Similarly, Mund et al. demonstrated that MRSA biofilms treated with *L. acidophilus* CFS underwent structural disruption and exhibited a marked increase in dead cells [26].

**Fig 6:**
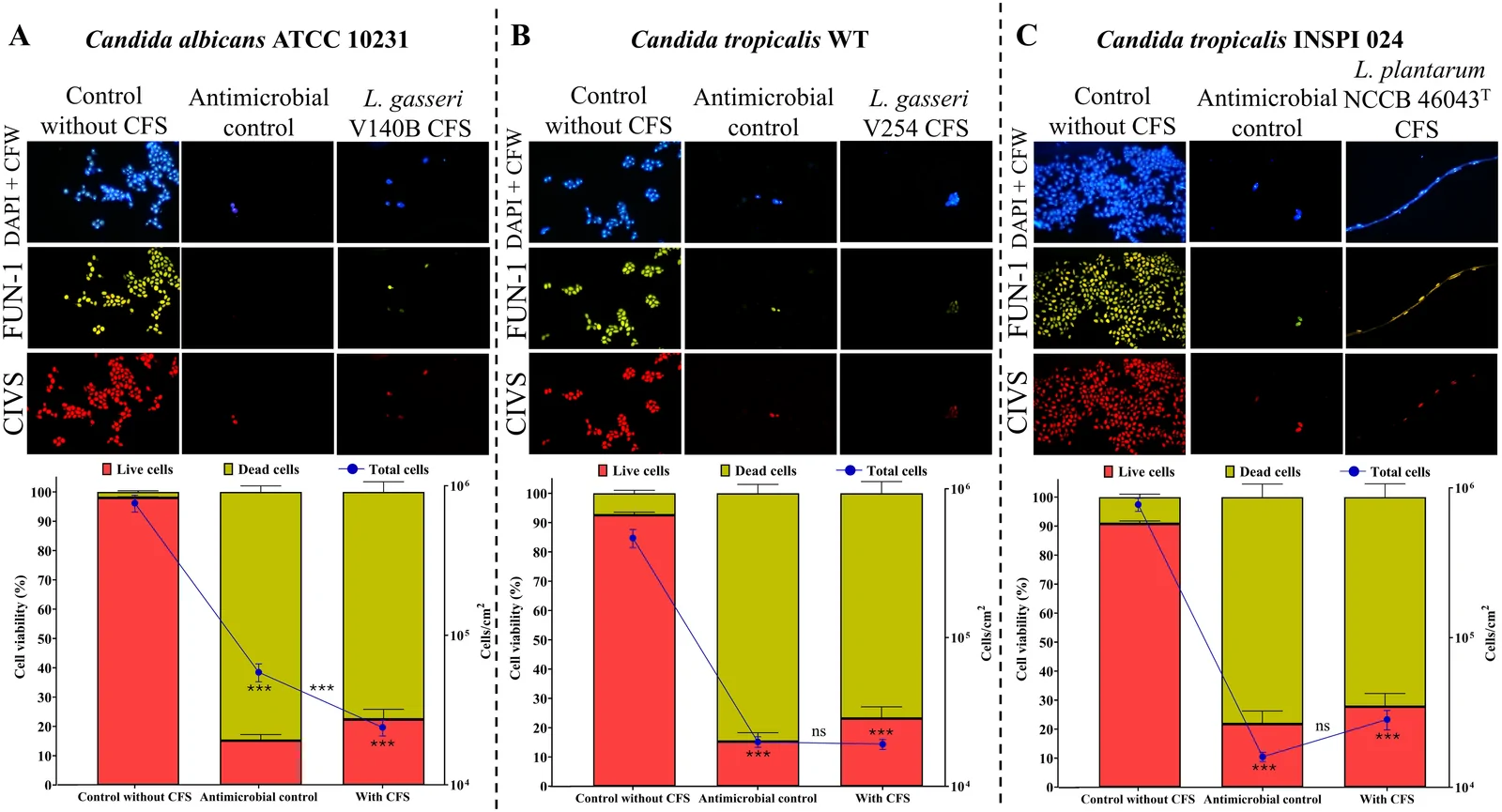
Microscopic visualization and cell viability of *Candida* strains treated with CFS. (A) *C. albicans* ATCC 10231; (B, C) *C. tropicalis* strains. DAPI stains total cellular DNA (blue), Calcofluor White M2R (CFW) labels cell wall chitin independent of metabolic state (blue), FUN-1 enters metabolically inactive or dead cells (yellowish green), while forming intravacuolar cylindrical structures (CIVS) in metabolically active cells. Bar graphs represent the percentage of live and dead cells, together with total cell density (cells/cm^2^) plotted on a logarithmic scale. Statistical differences were evaluated using the Wilcoxon test comparing untreated samples with antimicrobial controls and CFS treatments, as well as CFS versus antimicrobial controls (not significant, ns; *p* ≤ 0.05, *; *p* ≤ 0.01, **; *p* ≤ 0.001, ***). Bars represent mean values with standard error of the mean (SEM).

Regarding yeasts, fluorescence microscopy revealed a marked reduction in cell viability following treatment with CFS, with viable cells decreasing to approximately 25% across *Candida* species (Fig 6). For *C. albicans* ATCC 10231, treatment with CFS resulted in a significantly lower number of cells/cm² compared with the antifungal control, indicating a stronger antifungal effect (Fig 6A). In contrast, for *C. tropicalis* WT and *C. tropicalis* INSPI 024, CFS treatment achieved levels of cell reduction comparable to those observed with the antifungal control, with no statistically significant differences between these treatments (Fig 6B and 6C). Quantitative fluorescence microscopy data, including total cell counts and viability percentages, are provided in S9 Table.

These observations are consistent with previous studies reporting antifungal and antibiofilm effects of *Lactobacillus*-derived CFS against *Candida* species. Parolin et al. demonstrated that CFS from *L. crispatus* BC5 caused disruption and loss of biofilm architecture in *C. albicans* SO1 and *C. tropicalis* SO24, with fluorescence staining revealing few viable cells and a predominance of dead cells [49]. Similarly, Tan et al. reported that CLSM showed a reduction in mixed biofilms composed of *C. tropicalis* BF, *C. krusei* BF, and *C. parapsilosis* BF following treatment with CFS from *L. gasseri* BF and *L. rhamnosus* BF, together with an increased proportion of dead cells and poorly developed biofilm structure compared with untreated controls [50].

### Antimicrobial activity of CFS at neutral pH and after enzymatic degradation suggests the contribution of non-acidic metabolites

To explore the nature of the compounds responsible for the antimicrobial activity observed in previous assays, the contribution of acidic and non-acidic components of effective CFS was evaluated at the MIC concentration. The initial pH of the CFS ranged from 4.17 to 4.99; therefore, samples were neutralized to a pH 7.0 ± 0.1, and microbial growth inhibition was compared under acidic and neutral conditions.

**Fig 7:**
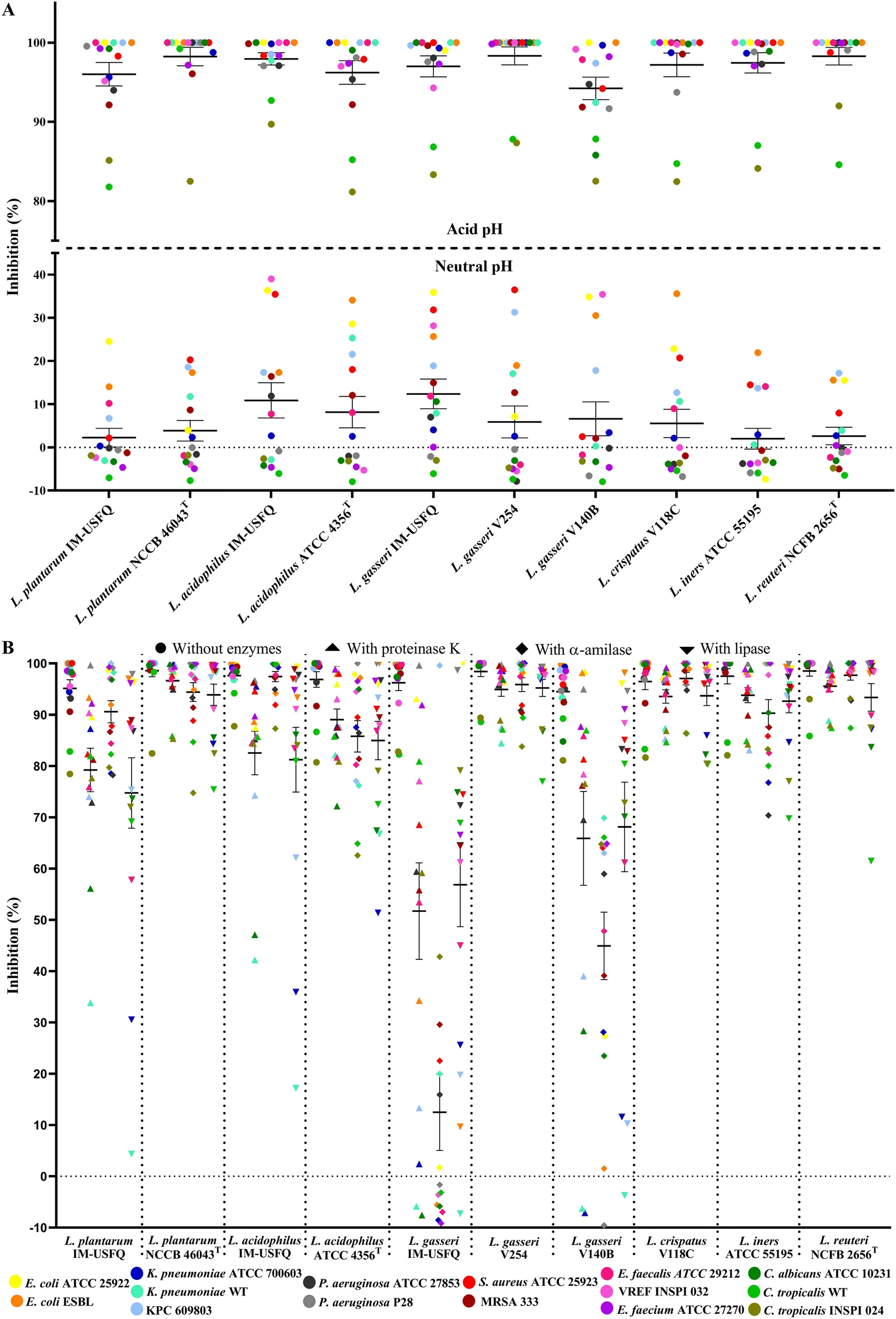
Antimicrobial activity of CFS following chemical modifications. (A) Effects of pH neutralization. (B) Effects of enzymatic degradation. Colored dots represent individual pathogens. Bars indicate median values with standard error of the mean (SEM).

Overall, pH neutralization resulted in a marked but partial reduction in antimicrobial activity. While inhibition values at the native acidic pH exceeded 80%, neutralized CFS showed inhibition levels ≤ 40% (Fig 7A). These results indicate that organic acidity is a major contributor to antimicrobial activity but also demonstrate that non-acidic components remain active and contribute to growth inhibition. Detailed inhibition percentages and statistical analyses are provided in S10 Table. Similar observations have been reported previously in the literature. Scillato et al. described increased microbial growth when CFS from *L. gasseri* 1A-TV, *L. fermentum* 18A-TV, and *L. crispatus* 35A-TV was neutralized from pH of 4.2 to 6.6–7.5, as reflected by increased OD_600/530 nm_ values [24]. Likewise, Khani et al. observed a decrease in inhibition of *S. aureus* to 19–54% following neutralization of CFS from *L. acidophilus*, *L. paracasei*, and *L. plantarum*, when compared with nearly complete inhibition at pH of 4 [51]. These results can also be viewed in the context of the recent study by Santarelli et al., who reported that the CFS of *L. crispatus* M247 exhibited antimicrobial activity against clinically relevant vaginal pathogens, and that this activity is due to the relative contribution of organic acids, proteins or peptides, carbohydrates, and lipids, and should be interpreted as a multifactorial and strain-dependent phenomenon [18].

To further assess the contribution of specific non-acidic fractions, effective CFS at the MIC concentration were subjected to enzymatic degradation using proteinase K, α-amylase, and lipase, targeting proteins/peptides, polysaccharides, and lipid components, respectively. Compared with untreated CFS, enzymatic treatment resulted in a partial or, in some cases, near-complete loss of antimicrobial activity (Fig 7B). It is important to note that enzymatic degradation assays provide only an indirect and preliminary indication of the biochemical nature of the active fractions. These assays suggest the possible contribution of protein components, carbohydrate-associated components, or lipid-related components, but they do not allow for the identification of antimicrobial molecules. This effect was particularly pronounced for CFS from *L. gasseri* IM-USFQ and *L. gasseri* V140B. In these samples, mean inhibition across all pathogens decreased to 52% and 65% after proteinase K treatment, 13% and 45% after α-amylase treatment, and 57% and 68% after lipase treatment, respectively. These findings suggest that the antimicrobial potential of CFS is multifactorial, involving not only organic acids but also enzymatically labile compounds, including proteins or peptides, carbohydrate-based molecules such as exopolysaccharides, and lipid-derived components. Detailed inhibition data and statistical significance values are reported in S11 Table. Consistent with this interpretation, Yang et al. demonstrated that the antimicrobial activity of *L. reuteri* AN417 CFS against *Porphyromonas gingivalis* strain BAA-308 was markedly reduced following enzymatic treatment, implicating fatty acids, sugars, and related compounds as active components [23]. Similarly, Arrioja-Bretón et al. reported that the anti-*Listeria* activity of *L. plantarum* NRRL B-4496 CFS was completely abolished after proteinase K treatment, supporting a central role for proteinaceous compounds in antimicrobial activity [25].

### Cell-type dependent responses observed in MTT and fluorescence assays

To evaluate the cytotoxicity potential of CFS, MTT assays were performed using HepG2 (hepatic epithelial) and RAW 264.7 (murine macrophage) cell lines exposed to CFS at 20 mg/mL and neutral pH. No statistically significant differences were observed between effective and ineffective CFS in HepG2 cells (*p* = 0.953), whereas a significant difference was detected in RAW 264.7 cells (*p* = 0.0280).

**Fig 8:**
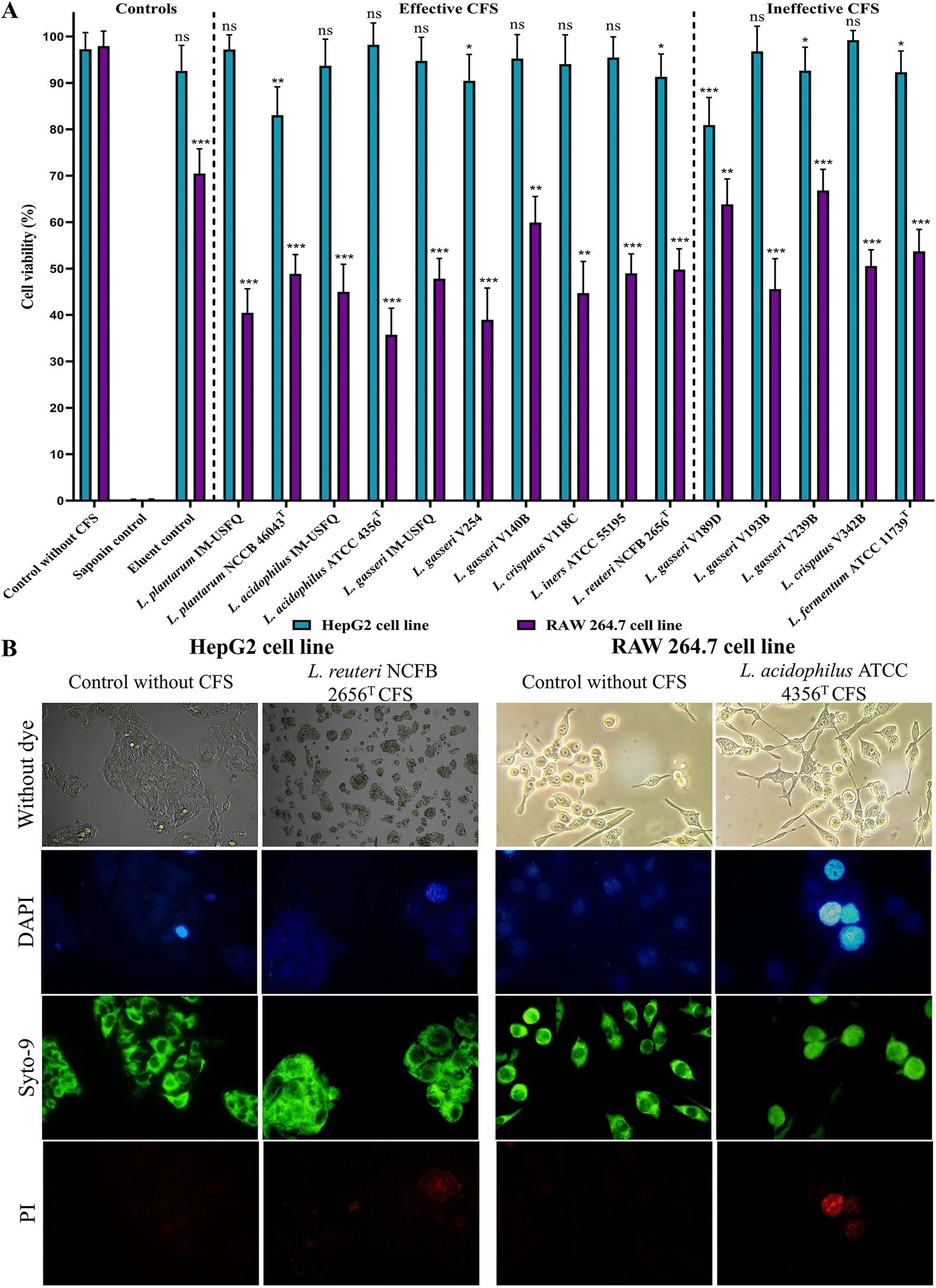
Cytotoxicity assessment of CFS. (A) Cell viability determined by MTT assay in HepG2 and RAW 264.7 cells. Bars represent mean percent cell viability with standard deviation (SD). (B) Fluorescence microscopic images of representative treatments showing DAPI-stained nuclei (blue), SYTO^TM^ 9-positive live cells (green), and propidium iodide (PI)-positive dead cells (red). Statistical differences were assessed using the Wilcoxon test relative to untreated controls or eluent controls (not significant, ns; *p* ≤ 0.05, *; *p* ≤ 0.01, **; *p* ≤ 0.001, ***).

The HepG2 cell line exhibited a high degree of resilience, with cell viability remaining largely unaffected, ranging between 81% and 99% across treatments. Statistically significant reductions were limited to specific CFS, notably *L. gasseri* V189D (81%) and *L. plantarum* NCCB 46043T (83%) (Fig 8A). This epithelial tolerance is consistent with previous findings reporting minimal cytotoxicity or even proliferative effects of *Lactobacillus*-derived postbiotics in intestinal epithelial models, such as HT-29 cells [51].

In contrast, RAW 264.7 macrophages displayed pronounced sensitivity, with a > 68% reduction in metabolic activity as measured by MTT. Importantly, a marked decrease in viability was also observed for the eluent control, an effect not observed in HepG2 cells, suggesting that macrophages possess an intrinsically higher sensitivity to vehicle composition and exposure conditions. Quantitative MTT data are provided in S12 Table.

Notably, the strong reduction in MTT signal observed in RAW 264.7 cells did not correlate with extensive cell death. Fluorescence microscopy revealed largely intact nuclei, preserved membrane integrity, and high overall viability, with structural damage confined to a minor subpopulation (Fig 8B). This apparent discrepancy may suggest a mismatch between MTT-derived metabolic activity and direct fluorescence-based indicators of membrane integrity and cell morphology. One possible explanation is that these findings may be compatible with changes in macrophage metabolic state, including immunometabolic reprogramming [52]. Such metabolic changes could affect mitochondrial reductive activity and, consequently, formazan production in the MTT assay, without necessarily reflecting proportional loss of cell viability [53]. This possibility is consistent with the increased filopodia formation and enhanced fluorescence signaling observed in RAW 264.7 cells following CFS exposure, although these qualitative observations are not sufficient to confirm cellular activation or exclude all forms of cytotoxic stress and this hypothesis requires further validation using complementary metabolic and viability assays.

Comparable effects have been reported by Nanjundaiah et al., who demonstrated that conditioned medium from *L. rhamnosus* GG modulates macrophage bactericidal activity and immune signaling without inducing significant cytotoxicity [53]. Taken together, these findings suggest that exposure to CFS may alter the metabolic activity and morphology of macrophages without causing extensive membrane damage under the conditions evaluated. However, further studies are needed to distinguish between actual cytotoxicity, metabolic modulation, or direct interference with the MTT assay.

The persistence of biological effects after pH neutralization further supports the involvement of non-acidic metabolites in CFS bioactivity. Indeed, Luo et al. identified fatty acids, sugars, and other metabolites produced by *L. reuteri* AN417 that exert biological activity independently of lactic acid [54]. In contrast to the study by Abbasi et al., which reported pronounced cytotoxic and pro-apoptotic effects of CFS in cancer models [55], the outcomes observed here emphasize that the biological outcome of *Lactobacillus* secretomes is highly context- and cell type-dependent.

Overall, the differences in response observed between HepG2 and RAW 264.7 cells suggest that the effects of CFS are cell type-dependent. However, the reduction in MTT signal in RAW 264.7 cells should be interpreted with caution, as it could reflect altered metabolic activity, assay interference, or cellular stress, and not solely direct cytotoxicity. A major limitation of this study is that all *Lactobacillus* strains evaluated were derived from the vaginal microbiota. This selection was determined by the availability of strains from microbial collections and previous projects; however, it should be acknowledged that the common origin of the isolates may introduce bias in the interpretation of the results. Antimicrobial activity, metabolic composition, and functional potential of CFS may be influenced by the niche of origin, local selective pressures, interaction with the host, and strain-specific variability. An additional limitation of this study is that the chemical characterization of CFS was exploratory and focused on specific groups of compounds; therefore, the lack of a more in-depth molecular characterization using techniques such as LC-MS/MS, metabolomics, or proteomics limits the ability to attribute bioactivity to specific compounds, such as bacteriocins, lipopeptides, fatty acids, or other secondary metabolites.

In conclusion, the evaluated CFS demonstrated a strong capacity to inhibit a broad spectrum of both susceptible and multidrug-resistant pathogens at relatively low concentrations. Chemical and functional analyses indicate that this antimicrobial activity is not exclusively driven by acidic components, but instead involves additional bioactive metabolites, including peptides, proteins, carbohydrates, exopolysaccharides, lipids, and possibly other molecular classes. These findings highlight the multifactorial nature of the antimicrobial mechanisms associated with *Lactobacillus*-derived CFS. Despite these promising results, further in-depth chemical, metabolomic, and genomic analyses are required to precisely identify and characterize metabolites responsible for the observed bioactivity and to assess their potential biopharmaceutical or therapeutic applications. Finally, the cytotoxic assays revealed a cell type-dependent response, with macrophages exhibiting greater sensitivity than hepatic epithelial cells. It is important to note that the reduction in MTT signal observed in macrophages should be interpreted with caution, as it could reflect metabolic changes, assay-related interference, or cellular stress; therefore, further studies are needed to define the cytotoxic effect.

## Materials and methods

### Identification of microbial strains and culture conditions

Microbial identification was performed using independent strategies. *Lactobacillus* strains were confirmed by PCR, while pathogens, either previously characterized or from reference strains, were confirmed by MALDI-TOF MS. The application of these methods is detailed below. Thirty *Lactobacillus* strains from different microbial collections and previous studies [56–58] were selected (S1 Table), and their identities were confirmed through PCR amplification of the V3-V4 regions of the 16S rRNA gene. DNA was extracted and PCR amplification was performed using primers detailed in S2 Table, as realized in previous studies [59,60]. Briefly, PCR conditions included an initial denaturation step at 95 °C for 2 minutes, followed by 35 cycles of denaturation at 94 °C for 20 seconds, annealing at 54 °C for 20 seconds, extension at 72 °C for 40 seconds, and a final extension at 72 °C for 5 minutes. Identification was performed by visualizing the amplicons on a 1.5% agarose gel. Taxonomic confirmation using this methodology has previously been used with high concordance at the genus level [61,62], but with lower resolution in the differentiation between phylogenetically closely related species [63].

Fifteen pathogenic microorganisms from different microbial collections and previous studies [60,64–77] were selected (S3 Table) and their identities were confirmed using matrix-assisted laser desorption/ionization time-of-flight mass spectrometry (MALDI-TOF MS) with a MALDI Biotyper system (Bruker Daltonics, Germany). Each microbial colony was distributed in one well of a steel plate and coated with 1 µL of α-cyano-4-hydroxycinnamic acid (HCCA) matrix solution. After drying at room temperature, the samples were analyzed, and the resulting spectra were compared with the MBT Compass Explorer software database (v3.7) (Bruker Daltonics, Germany), as previously described [31,78].

All strains were maintained in appropriate culture media and specific conditions. *Lactobacillus* strains were cultured by streaking from glycerol at 15% stock cultures on Man, Rogosa, and Sharpe (MRS) agar (Becton, Dickinson and Company, USA) and incubated at 37 °C under microaerophilic conditions for 24–48 hours. Most pathogens were isolated on Tryptic Soy Agar (TSA) (Becton, Dickinson and Company, USA) and incubated at 37 °C in aerobic conditions except for *Candida* species, which were cultured on Sabouraud Dextrose agar (Becton, Dickinson and Company, USA) under the same conditions.

### Cell-Free Supernatant (CFS) preparation

The CFS preparation was based on previous studies by Sornsenee et al. [27] and Yang et al. [23] with slight modifications. Briefly, each strain of *Lactobacillus* was cultured in 50 mL of MRS broth (Becton, Dickinson and Company, USA) at 37 °C for 24 hours under microaerophilic conditions, then centrifuged at 5,000 rpm for 10 minutes and the supernatant was filtered using Durapore® 0.22 µm polyvinylidene fluoride (PVDF) membrane filters (MilliporeSigma, USA) to completely remove the bacterial cells. The CFS was lyophilized in Freeze Dryer BK-FD10P (Biobase Biodustry, China) and stored at −20 °C until further use.

### Quantification of carboxylic acids by High-Performance Liquid Chromatography (HPLC)

Citric acid, lactic acid, malic acid, and oxalic acid of the CFS were quantified by HPLC according to the method described by Nour et al. [79] with slight modifications. Briefly, the filtered CFS was injected into an Agilent Technologies Series 1260 HPLC system (Agilent Technologies, USA) equipped with a diode array detector (DAD), and an Eclipse Plus C18 column (5 µm 4.6 x 250 mm). The mobile phase used was potassium dihydrogen phosphate (KH_2_PO_4_) 50 mM at pH 2.8 at a flow rate of 0.7 mL/min with isocratic elution mode and detection wavelength at 210 nm. Calibration curves for each standard of carboxylic acid were used for quantification and the lowest calibration level evaluated was 5 ppm for citric, lactic and oxalic acids and 10 ppm for malic acid (Merck, Germany) (S1 File). Estimated limit of quantitation (LOQ) values were 0.685, 1.525, 5.972 and 0.0465 ppm for citric, lactic, malic, and oxalic acids, respectively. These carboxylic acids were included as part of an initial profile of organic acids detectable by HPLC.

### Estimation of free amino acids by ninhydrin-cadmium colorimetric method

The concentrations of leucine and proline in the CFS were determined using the ninhydrin-cadmium colorimetric method [80]. Leucine and proline were selected as representative markers of free amino acids to estimate the amino acid fraction of CFS and indirectly assess proteolytic activity. The working solution was prepared with 0.8 g of ninhydrin, 80 mL of ethanol (99.5%), 10 mL acetic acid, and 1 g of cadmium chloride (Merck, Germany), then 1 mL of each CFS at 4 mg/mL was mixed with 2 mL of working solution. This mixture was heated at 84 °C for 5 minutes and then cooled using a cold block and absorbance was measured in UV-VIS Spectrophotometer i3 (Hanon Instruments, China) at 507 nm. Standard curves (S1 File) were used to calculate the concentration of each N-amino (S4 Table).

### Determination of total protein concentration by Bradford method

Total protein concentration in the CFS was determined using the Bradford method as originally described by Bradford [81]. Briefly, 50 µL of CFS at 400 mg/mL was mixed with 500 µL of Bradford reagent (Thermo Fisher Scientific Inc., USA), incubated at room temperature for 2 minutes and absorbance was immediately measured at 595 nm. A standard curve (S1 File) was generated using bovine serum albumin (BSA) (Gibco, USA) ranging 0.1–1 mg/mL concentration to calculate the total protein concentration (mg/g; see S4 Table).

### Antimicrobial activity screening

The antimicrobial activity of the thirty *Lactobacillus* CFS was evaluated against fifteen pathogens (including multidrug-resistant pathogenic strains) using the microdilution method in 96-well plates to determine the minimum inhibitory concentration (MIC) and minimum bactericidal or fungicidal concentration (MBC or MFC) following the guidelines of the Clinical and Laboratory Standards Institute (CLSI) and Wiegand et al. [82]. Because the CFS samples were lyophilized, the concentrations evaluated could be standardized as dry mass of CFS per final assay volume, expressed in mg/mL. In MIC assays, dilutions of the lyophilized CFS were prepared to evaluate antimicrobial activity at the final concentration of 10, 20, 40, 60, and 80 mg/mL using autoclaved distilled water [83]. Subsequently, 10 µL of 20x concentrated CFS was mixed with 190 µL of pathogen suspension, previously prepared in Mueller-Hinton broth to a final concentration of 5 x 10^5^ colony-forming units (CFU)/mL. Plates were incubated at 37 °C for 18–24 hours and absorbance was measured at 570 nm in the spectrophotometer ELx808IU (Bio Tek Instruments Inc., USA). Each assay was conducted in triplicate on three independent experiments including antimicrobial control (ciprofloxacin, ampicillin, or fluconazole), blank or sterility control (CFS and culture medium without the microorganism), and positive control without CFS (culture medium with the microorganism). The following formula was used to calculate the percentage of inhibition:


$$\begin{array}{c} Inhibition\;(\%)=\left(\text{1}-\frac{{OD}_{with\;CFS}-{OD}_{blank\;control}}{{OD}_{control\;without\;CFS}-{OD}_{blank\;control}}\right)x\text{100} \end{array}$$ (1)

MIC was determined by identifying the lowest concentration of CFS at which significant inhibition of pathogen growth was observed (S5 and S6 Tables). CFS were classified as effective (≤ 10–20 mg/mL) or ineffective (≥ 60–80 mg/mL; S1 Fig). The classification of CFS as effective or ineffective was used solely as an operational category within the experimental conditions of this study to distinguish preparations with higher or lower antimicrobial activity under standardized conditions. This classification was based on the MIC and MBC or MFC values obtained in the initial screening and was subsequently cross-checked with complementary variables, including biomass, log (CFU/mL) counts, fluorescence microscopy, pH neutralization, enzymatic treatments, and partial chemical characterization.

The resazurin reagent (Acros Organics, China) was used to determine the MBC or MFC, following the method described by Elshikh et al. with slight modifications [84]. Briefly, 30 µL of resazurin was added to each well of the plate and incubated at 37 °C for 3 hours. After incubation, the resulting color of the treated wells was compared with the controls, and the MBC or MFC was defined as the lowest concentration showing a color different from the positive control without CFS (i.e., typically purple or bluish indicating absence of microbial metabolic activity).

### Assessment of bactericidal effect by Colony Forming Unit (CFU) counting

CFU were quantified to compare the bactericidal effect of the 10 most effective CFS and 5 least effective CFS against all pathogens. The assay was conducted exclusively at the MIC of each CFS against each pathogen (S5 and S6 Tables), previously determined in the microdilution assay. At least nine individual suspensions of each sample (triplicate on three independent experiments) were used in serial dilutions by adding 100 µL of sample in 900 µL of sterile saline solution. Each dilution (1^00^, 10^−1^, 10^−2^, 10^−3^, 10^−4^, 10^−5^, 10^−6^) was mixed thoroughly in the vortex and three drops of 10 µL were plated onto TSA and were spread across the surface of the agar to obtain well-isolated, countable colonies, avoiding contact with the plate walls, in triplicate, and incubated at 37 °C for 24 hours. The next day, dilutions yielding between 25 and 250 colonies were selected for counting to calculate log (CFU/mL) (S7 and S8 Tables) and obtain statistically reliable results according to previous studies [85,86].

### Fluorescence staining and microscopic analysis

Bacteria samples were stained with LIVE/DEAD BacLight^TM^ Bacterial Viability Kit (Thermo Fisher Scientific, USA) which contains SYTO^TM^ 9 to visualize live cells and propidium iodide (PI) to visualize dead cells, along with 4´,6-diamidino-2-phenylindole, dilactate (DAPI) (Thermo Fisher Scientific, USA) to visualize total cells, as previously realized [87]. On the other hand, yeast samples were stained with DAPI and LIVE/DEAD™ Yeast Viability Kit (Thermo Fisher Scientific, USA) which contains FUN-1 to visualize live cells and Calcofluor White M2R to visualize all cells, as previously described [88]. The staining was performed according to the manufacturer´s instructions with minor modifications. Briefly, 100 µL of each sample (triplicate on three independent experiments of each pathogen with its control without CFS and treated sample with a single CFS and antimicrobial control at the MIC) was placed in an Eppendorf tube and centrifuged at 10,000 rpm for 10 minutes. The supernatant was removed and the cells were resuspended and mixed in 20 µL of PBS. 3 µL of the LIVE/DEAD dilution (1:10) was added and incubated for 15 minutes at room temperature in complete darkness. Then, 3 µL of the DAPI dilution (1:10) was added and incubated for 15 minutes at room temperature in complete darkness. Immediately, 2 µL of mounting solution (Thermo Fisher Scientific, USA) with 5 µL of the stained solution was placed on a slide, covered with a coverslip, and waited 5–10 minutes for the cells to stabilize and immobilize. Immersion oil was placed on the coverslip and epifluorescence microscopy was carried out using an Olympus BX50F4 epifluorescence microscope (Olympus Optical Corporation, Japan) equipped with a 100x objective. A total of fifteen fields were captured with three different filters for each stain from evenly distributed and random microscopic fields [89] with AmScope Digital Camera MU633-FL (AmScope, USA) and digitalized with AmScope software (v3.7). The number of cells per field was counted with a pipeline designed in the CellProfiler software (v4.2.6) [90] and the number of cells/cm^2^ and percentage of live cells were reported for subsequent statistical analysis (S9 Table).

### pH neutralization of CFS and effects on antimicrobial activity

The antimicrobial contribution of acid metabolites in CFS was evaluated by measuring the pH of each CFS at the MIC with the calibrated Milwaukee Mi 151 pH meter (Milwaukee Instruments Inc., USA). Subsequently, the pH was adjusted to neutrality (pH 7 ± 0.1) using sterile 10 M NaOH. Microdilution assays were performed again with both acid and neutralized CFS against all pathogens. The microbial growth inhibition percentages were compared to determine if the antimicrobial activity persisted or decreased after neutralization (S10 Table), as described in other studies [24,25,51].

### CFS enzymatic degradation and effects on antimicrobial activity

To evaluate the biochemical nature of the antimicrobial compounds in CFS and selectively degrade proteins, polysaccharides, and lipids, the enzymes proteinase K, α-amylase, and lipase were used following the method described by Yang et al. [23] with slight modifications. Enzymes were prepared in sterile distilled water, the same solvent used for CFS reconstitution. Each CFS was diluted to its previously determined MIC and treated independently with proteinase K (1 mg/mL) (Thermo Fisher Scientific, Germany), α-amylase (150 U/mg) (Sigma-Aldrich Corporation, Denmark), and lipase (700 U/mg) (Sigma-Aldrich Corporation, Japan). Immediately, the mixtures were incubated at 37 °C without agitation for 3 hours to allow enzymatic degradation, then inactivated by heating the samples at 95 °C for 3 minutes and centrifuged at 1,000 rpm for 10 minutes to obtain the supernatants. Antimicrobial activity was determined by microdilution assay against all pathogens, and the results were expressed as a percentage of growth inhibition and each enzyme-treated CFS was compared with untreated CFS at MIC (S11 Table).

### MTT assay for evaluation of CFS cytotoxicity

CFS cytotoxicity was evaluated using (3-(4,5-dimethylthiazol-2-yl)-2,5-diphenyltetrazolium bromide) MTT colorimetric assay with HepG2 and RAW 264.7 cells that were seeded at density of 6,000 cells/well in 96-well plates in 190 µL of supplemented MEM (Sigma-Aldrich Corporation, Germany) and DMEM (Gibco, USA) medium, respectively. After 12 hours of incubation at 37 °C in a humidified 5% CO_2_ atmosphere, the cells were treated with 10 µL of CFS at a final concentration of 20 mg/mL and neutral pH, only media (control without CFS), saponin (4 mg/mL, positive control) or autoclaved distilled water (eluent control) and incubated for 72 hours under the same conditions. Immediately, 20 µL of MTT solution (Sigma-Aldrich Corporation, USA) were placed in each well at a final concentration of 0.45 mg/mL and plates were incubated at 37 °C for 2 hours protected from light. Following incubation, plates were centrifuged at 4000 rpm for 10 minutes, the supernatant was carefully removed and 100 µL of DMSO (Sigma-Aldrich Corporation, USA) was added to solubilize the formazan crystals. Absorbance was measured at 570 nm (test wavelength) and 630 nm (reference wavelength) using the spectrophotometer ELx808IU (BioTek Instruments Inc., USA). The percentage of cell viability was compared (S12 Table), as reported in other studies [25,51].

### Fluorescence imaging of cytotoxic effects

To visualize CFS-treated cells by fluorescence microscopy, HepG2 and RAW 264.7 cells were seeded at a density of 2 x 10^5^ cells/well in 6-well plates containing sterile coverslips placed at the bottom of each well. After cell adherence, the cells were incubated with CFS at 20 mg/mL and neutral pH or with the corresponding controls at 37 °C in a humidified 5% CO_2_ atmosphere. After incubation, the culture medium was removed, and cells were washed once with sterile PBS (1x). 150 µL of staining solution (3:2000) of LIVE/DEAD BacLight^TM^ Bacterial Viability Kit (Thermo Fisher Scientific, USA) was added to each well and incubated at room temperature for 15 minutes in the dark, followed by one wash with PBS (1x). 150 µL of DAPI (Thermo Fisher Scientific, USA) staining solution (3:2000) was added and incubated at room temperature for 15 minutes, also protected from light. After a final PBS wash, the coverslips were carefully removed and mounted on glass slides for observation under the Olympus BX50F4 epifluorescence microscope (Olympus Optical Corporation, Japan). Fluorescence images were captured with AmScope Digital Camera MU633-FL (AmScope, USA) and digitalized with AmScope software (v3.7) to evaluate indicators of cytotoxicity such as cell morphology, membrane integrity, nuclear condensation, and differential staining patterns.

### Statistical and graphical analysis

Data were analyzed using RStudio software (v4.2.2) with several R packages (“ggpubr”, “rstatix”, “openxlsx”, and “tidyverse”) [91]. Comparisons between two independent groups were performed using the Wilcoxon rank-sum test, a nonparametric method appropriate for small sample sizes, non-normally distributed data, and variables that do not meet parametric assumptions. This test was applied when observations were independent between groups, such as comparisons of cellular responses between effective and ineffective CFS within each cell line. A *p* value ≤ 0.05 was considered statistically significant. Differences between effective and ineffective CFS in antimicrobial assays were assessed using the paired Wilcoxon signed-rank test when the same pathogen panel was evaluated across both CFS categories. In these analyses, each pathogen was treated as a paired observational unit to account for pathogen-specific variability in susceptibility. MIC and MBC or MFC values exceeding the highest tested concentration were recorded as the maximum tested value for statistical analysis, allowing standardized comparison across treatments. The statistical approach was selected to provide a conservative comparison of CFS activity under the experimental conditions of this study. Data visualizations, including boxplots and bar plots, were generated using GraphPad Prism (v8.0.1) [92].

## Supporting information

S1 Table Description of *Lactobacillus* strains used for Cell-Free Supernatant (CFS) production. (DOCX)

S2 Table Primers used for *Lactobacillus* strain identification. (DOCX)

S3 Table Description and MALDI-TOF identification of pathogenic microorganisms. (DOCX)

S4 Table Metabolite concentrations in CFS from *Lactobacillus* strains. (DOCX)

S5 Table Comparison of MIC and MBC or MFC values between effective CFS and antimicrobial control. (DOCX)

S6 Table Comparison of MIC and MBC or MFC values among ineffective CFS. (DOCX)

S7 Table Bacterial growth and viability in response to effective CFS and standard antimicrobials. (DOCX)

S8 Table Bacterial growth and viability in response to ineffective CFS. (DOCX)

S9 Table Quantitative fluorescence microscopy data. (DOCX)

S10 Table Effects of pH neutralization on the antimicrobial activity of CFS. (DOCX)

S11 Table Antimicrobial activity of CFS after enzymatic degradation. (DOCX)

S12 Table Cell viability response to CFS as assessed by MTT assay. (DOCX)

S1 Fig Comparative heatmap of CFS antimicrobial potential based on MIC values. (TIF)

S1 File Calibration curves. (XLSX)
