# Supplementary material for: Cell-Free but potent: Antimicrobial potential of Lactobacillus supernatants against multidrug-resistant pathogens
Source: PLoS One. 2026 Aug 25;21(8):e0356730. doi: 10.1371/journal.pone.0356730 (PMC13505906; doi:10.1371/journal.pone.0356730)
Supplement: Supplementary file 1 [file pone.0356730.s001.docx]

**Supplementary Table S1.** Description of *Lactobacillus* strains used for Cell-Free Supernatant (CFS) production.

| **Microbial strain** | **Provided by** | **Isolation source / Collection** | **References** |
| --- | --- | --- | --- |
| *Lactiplantibacillus plantarum* IM-USFQ | IM-USFQ | Clinical isolate from a vaginal sample in Ecuador | [55] |
| *Lactiplantibacillus plantarum* NCCB 46043^T^ | University of Ghent | Netherlands Culture Collection of Bacteria | [56] |
| *Lactobacillus acidophilus* IM-USFQ | IM-USFQ | Clinical isolate from a vaginal sample in Ecuador | [55] |
| *Lactobacillus acidophilus* ATCC 4356^T^ | University of Ghent | American Type Culture Collection | [56] |
| *Lactobacillus gasseri* IM-USFQ | IM-USFQ | Clinical isolate from a vaginal sample in Ecuador | [55] |
| *Lactobacillus gasseri* V130B | IM-USFQ | Clinical isolate from a vaginal sample in Ecuador - Coinfection | [55] |
| *Lactobacillus gasseri* V140B | IM-USFQ | Clinical isolate from a vaginal sample in Ecuador - Healthy microbiota | [55] |
| *Lactobacillus gasseri* V239A | IM-USFQ | Clinical isolate from a vaginal sample in Ecuador - Healthy microbiota | [55] |
| *Lactobacillus gasseri* V254 | IM-USFQ | Clinical isolate from a vaginal sample in Ecuador - Healthy microbiota | [55] |
| *Lactobacillus gasseri* V189C | IM-USFQ | Clinical isolate from a vaginal sample in Ecuador - Aerobic vaginitis | [55] |
| *Lactobacillus gasseri* V189D | IM-USFQ | Clinical isolate from a vaginal sample in Ecuador - Aerobic vaginitis | [55] |
| *Lactobacillus gasseri* V193B | IM-USFQ | Clinical isolate from a vaginal sample in Ecuador - Intermediate Microbiota | [55] |
| *Lactobacillus gasseri* V239B | IM-USFQ | Clinical isolate from a vaginal sample in Ecuador - Healthy microbiota | [55] |
| *Lactobacillus gasseri* V245A | IM-USFQ | Clinical isolate from a vaginal sample in Ecuador | [55] |
| *Lactobacillus gasseri* V251C | IM-USFQ | Clinical isolate from a vaginal sample in Ecuador - Healthy microbiota | [55] |
| *Lactobacillus crispatus* ATCC 33820^T^ | University of Ghent | American Type Culture Collection | [56] |
| *Lactobacillus crispatus* V118C | IM-USFQ | Clinical isolate from a vaginal sample in Ecuador | [55] |
| *Lactobacillus crispatus* V217D | IM-USFQ | Clinical isolate from a vaginal sample in Ecuador - Healthy microbiota | [55] |
| *Lactobacillus crispatus* V247A | IM-USFQ | Clinical isolate from a vaginal sample in Ecuador - Healthy microbiota | [55] |
| *Lactobacillus crispatus* V342B | IM-USFQ | Clinical isolate from a vaginal sample in Ecuador - Healthy microbiota | [55] |
| *Lactobacillus iners* ATCC 55195 | Universidade do Minho | American Type Culture Collection | [57] |
| *Limosilactobacillus reuteri* NCFB 2656^T^ | University of Ghent | United Kingdom National Collection of Food Bacteria | [56] |
| *Limosilactobacillus fermentum* ATCC 11739^T^ | University of Ghent | American Type Culture Collection | [56] |
| *Lactobacillus jensenii* IM-USFQ | IM-USFQ | Clinical isolate from a vaginal sample in Ecuador | [55] |
| *Lactobacillus* sp*.* V156A | IM-USFQ | Clinical isolate from a vaginal sample in Ecuador - Healthy microbiota | [55] |
| *Lactobacillus* sp*.* V174D | IM-USFQ | Clinical isolate from a vaginal sample in Ecuador - Healthy microbiota | [55] |
| *Lactobacillus* sp. V221B | IM-USFQ | Clinical isolate from a vaginal sample in Ecuador - Healthy microbiota | [55] |
| *Lactobacillus* sp. V280E | IM-USFQ | Clinical isolate from a vaginal sample in Ecuador - Healthy microbiota | [55] |
| *Lactobacillus* sp. V310C | IM-USFQ | Clinical isolate from a vaginal sample in Ecuador - Healthy microbiota | [55] |
| *Lactobacillus* sp. 102B | IM-USFQ | Clinical isolate from a vaginal sample in Ecuador | [55] |

IM-USFQ: Universidad San Francisco de Quito - Institute of Microbiology; ^T^: Type strain (reference strain used to define the species).
