# Supplementary material for: Cell-Free but potent: Antimicrobial potential of Lactobacillus supernatants against multidrug-resistant pathogens
Source: PLoS One. 2026 Aug 25;21(8):e0356730. doi: 10.1371/journal.pone.0356730 (PMC13505906; doi:10.1371/journal.pone.0356730)
Supplement: Supplementary file 2 [file pone.0356730.s002.docx]

**Supplementary Table S2.** Primers used for *Lactobacillus* strain identification.

| **Strains** | **Primers Forward** | **Primers Reverse** | **Amplicon size (bp)** | **DNA target** | **References** |
| --- | --- | --- | --- | --- | --- |
| *Lactobacillus* sp. | TGGAAACAGRTGCTAATACCG | GTCCATTGTGGAAGATTCCC | 232 | *16S* rRNA | [29,30] |
| *L. acidophilus* | TCAATCAAAGGAAGAGCGAG | CGCTCGCAATTTCGCTTA | 222 |  |  |
| *L. crispatus* | TTACTTCGGTAATGACGTTA | GGAACTTTGTATCTCTACAA | 966 |  |  |
| *L. fermentum* | ACTAACTTGACTGATCTACGA | TTCACTGCTCAAGTAATCATC | 191 |  |  |
| *L. gasseri* | TCGAGCGAGCTTGCCTAGATGAA | CGCGGCGTTGCTCCATCAGA | 372 |  |  |
| *L. iners* | GTCTGCCTTGAAGATCGG | ACAGTTGATAGGCATCATC | 158 |  |  |
| *L. jensenii* | AGTCGAGCGAGCTTGCCTATAGAAG | CTCACCCATCCGCCGCTAGCT | 70 |  |  |
| *L. plantarum* | CCTGAACTGAGAGAATTTGA | ATTCATAGTCTAGTTGGAGGT | 248 |  |  |
| *L. reuteri* | CAGACAATCTTTGATTGTTTAG | GCTTGTTGGTTTGGGCTCTTC | 303 |  |  |
