# Supplementary material for: Cell-Free but potent: Antimicrobial potential of Lactobacillus supernatants against multidrug-resistant pathogens
Source: PLoS One. 2026 Aug 25;21(8):e0356730. doi: 10.1371/journal.pone.0356730 (PMC13505906; doi:10.1371/journal.pone.0356730)
Supplement: Supplementary file 3 [file pone.0356730.s003.docx]

**Supplementary Table S3.** Description and MALDI-TOF identification of pathogenic microorganisms.

| **Microbial strain** | **Obtained from** | **Isolation source / Collection** | **Resistance profile** | **MALDI-TOF MS** | | **References** |
| --- | --- | --- | --- | --- | --- | --- |
|  |  |  |  | **Organism (best match)** | **Score value** |  |
| *Escherichia coli* ATCC 25922 | IM-USFQ | American Type Culture Collection | Susceptible | *Escherichia coli* ATCC 25922 | 2.30 | [63] |
| *Escherichia coli* extended-spectrum beta-lactamases | IM-USFQ | Clinical isolate in Ecuador | MDR | *Escherichia coli* DSM 1103 | 2.39 | [64,65] |
| *Klebsiella pneumoniae* ATCC 700603 | IM-USFQ | American Type Culture Collection | ESBL | *Klebsiella pneumoniae* subsp. *pneumoniae* DSM 30104^T^ | 2.28 | [66] |
| *Klebsiella pneumoniae* WT | IM-USFQ | Environmental Isolation in Ecuador | Susceptible | *Klebsiella pneumoniae* subsp*. ozaenae* DSM 16358^T^ | 2.08 | [59,67] |
| *Klebsiella pneumoniae* carbapenemase-producing 609803 | UDLA | Clinical isolate in Ecuador | MDR | *Klebsiella pneumoniae* ssp*. pneumoniae* 9295_1_CHB typed as KPC positive | 2.25 | [68] |
| *Pseudomonas aeruginosa* ATCC 27853 | IM-USFQ | American Type Culture Collection | Susceptible | *Pseudomonas aeruginosa* ATCC 27853 | 2.23 | [69] |
| *Pseudomonas aeruginosa* P28 | UDLA | Clinical isolate in Ecuador | MDR | *Pseudomonas aeruginosa* ATCC 27853 | 2.22 | [70] |
| *Staphylococcus aureus* ATCC 25923 | IM-USFQ | American Type Culture Collection | Susceptible | *Staphylococcus aureus* ATCC 25923 | 2.18 | [71] |
| Methicillin-resistant *Staphylococcus aureus* 333 | UDLA | Clinical isolate in Ecuador | MDR | *Staphylococcus aureus* ATCC 33591 typed as MRSA | 2.33 | [72] |
| *Enterococcus faecalis* ATCC 29212 | IM-USFQ | American Type Culture Collection | Susceptible | *Enterococcus faecalis* ATCC 29212 | 2.25 | [73] |
| Vancomycin-resistant *Enterococcus faecalis* INSPI 032 | INSPI | Clinical isolate in Ecuador | MDR | *Enterococcus faecalis* 20247_4 CHB | 2.18 | [74] |
| *Enterococcus faecium* ATCC 27270 | IM-USFQ | American Type Culture Collection | Susceptible | *Enterococcus faecium* DSM 2146 | 2.23 | [75] |
| *Candida albicans* ATCC 10231 | IM-USFQ | American Type Culture Collection | Susceptible | *Candida albicans* DSM 1665 | 2.27 | [76] |
| *Candida tropicalis* WT | IM-USFQ | Environmental Isolation in Ecuador | Susceptible | *Candida tropicalis* DSM 5991 | 2.09 | [59] |
| *Candida tropicalis* INSPI 024 | INSPI | Clinical isolate in Ecuador | MDR | *Candida tropicalis* CBS 2322 | 2.06 | [74] |

IM-USFQ: Universidad San Francisco de Quito - Institute of Microbiology; UDLA: Universidad de las Américas; INSPI: Instituto Nacional de Investigación en Salud Pública; MDR: Multi-drug resistance; ESBL: Extended-spectrum beta-lactamase. A score value between 2.00 - 3.00 in MALDI-TOF MS indicates high confidence and consistency in identification.
