# Supplementary material for: Cell-Free but potent: Antimicrobial potential of Lactobacillus supernatants against multidrug-resistant pathogens
Source: PLoS One. 2026 Aug 25;21(8):e0356730. doi: 10.1371/journal.pone.0356730 (PMC13505906; doi:10.1371/journal.pone.0356730)
Supplement: Supplementary file 4 [file pone.0356730.s004.docx]

**Supplementary Table S4.** Metabolite concentrations in CFS from *Lactobacillus* strains.

| **CFS** | **Concentration ± SD (mg/g CFS)** | | | | | |
| --- | --- | --- | --- | --- | --- | --- |
|  | **Citric acid** | **Lactic acid** | **Oxalic acid** | **Leucine** | **Proline** | **Total proteins** |
| *L. plantarum* IM-USFQ | 6.72 ± 0.30 | 248.48 ± 1.01 | 3.46 ± 0.03 | 2.43 ± 0.17 | 66.33 ±4.60 | 1.07 ± 0.05 |
| *L. plantarum* NCCB 46043^T^ | 6.64 ± 0.11 | 252.78 ± 0.31 | 3.50 ± 0.01 | 1.06 ± 0.02 | 29.74 ± 0.57 | 1.06 ± 0.03 |
| *L. acidophilus* IM-USFQ | 9.99 ± 0.22 | 107.42 ± 0.57 | 4.02 ± 0.01 | 2.17 ± 0.11 | 59.36 ± 2.84 | 1.10 ± 0.02 |
| *L. acidophilus* ATCC 4356^T^ | 8.55 ± 0.20 | 220.13 ± 0.53 | 3.66 ± 0.01 | 2.64 ± 0.07 | 71.94 ± 1.89 | 0.88 ± 0.11 |
| *L. gasseri* IM-USFQ | 11.10 ± 0.06 | 183.80 ± 1.09 | 3.96 ± 0.01 | 2.17 ± 0.03 | 59.36 ± 0.79 | 1.09 ± 0.03 |
| *L. gasseri* V130B | 4.27 ± 0.12 | 126.92 ± 2.53 | 2.67 ± 0.02 | 2.38 ± 0.16 | 65.12 ± 4.19 | 1.16 ± 0.10 |
| *L. gasseri* V140B | 8.75 ± 0.15 | 185.63 ± 0.44 | 2.17 ± 0.01 | 6.52 ± 0.07 | 175.20 ± 1.89 | 1.10 ± 0.04 |
| *L. gasseri* V239A | 7.45 ± 0.14 | 184.90 ± 0.81 | 1.85 ± 0.01 | 4.45 ± 0.09 | 120.12 ± 2.49 | 1.49 ± 0.07 |
| *L. gasseri* V254 | 11.96 ± 0.17 | 233.33 ± 1.01 | 2.78 ± 0.02 | 2.45 ± 0.03 | 66.94 ± 0.73 | 0.90 ± 0.09 |
| *L. gasseri* V189C | 7.05 ± 0.06 | 205.45 ± 1.22 | 2.30 ± 0.03 | 5.51 ± 0.02 | 148.38 ± 0.47 | 1.76 ± 0.03 |
| *L. gasseri* V189D | 9.23 ± 0.10 | 175.78 ± 0.86 | 2.59 ± 0.01 | 2.35 ± 0.05 | 64.21 ± 1.29 | 0.89 ± 0.12 |
| *L. gasseri* V193B | 0.17 ± 0.01 | 49.56 ± 0.91 | 2.00 ± 0.01 | 1.48 ± 0.04 | 40.95 ± 0.99 | 1.03 ± 0.06 |
| *L. gasseri* V239B | 0.17 ± 0.01 | 115.84 ± 1.04 | 2.74 ± 0.01 | 5.80 ± 0.03 | 156.03 ± 0.73 | 1.09 ± 0.05 |
| *L. gasseri* V245A | 0.17 ± 0.01 | 180.32 ± 3.09 | 4.37 ± 0.04 | 1.92 ± 0.06 | 52.77 ± 1.49 | 0.85 ± 0.08 |
| *L. gasseri* V251C | 10.73 ± 0.08 | 157.41 ± 0.75 | 3.97 ± 0.01 | 2.55 ± 0.05 | 69.67 ± 1.39 | 1.13 ± 0.05 |
| *L. crispatus* ATCC 33820^T^ | 0.17 ± 0.01 | 170.88 ± 0.21 | 3.96 ± 0.01 | 5.97 ± 0.10 | 160.65 ± 2.62 | 1.06 ± 0.08 |
| *L. crispatus* V118C | 12.28 ± 0.08 | 247.27 ± 2.40 | 3.18 ± 0.05 | 2.69 ± 0.07 | 73.23 ± 1.78 | 1.10 ± 0.01 |
| *L. crispatus* V217D | 7.63 ± 0.12 | 120.36 ± 0.56 | 2.29 ± 0.01 | 3.73 ± 0.06 | 100.88 ± 1.60 | 0.89 ± 0.01 |
| *L. crispatus* V247A | 12.03 ± 0.05 | 186.99 ± 1.08 | 4.44 ± 0.01 | 1.94 ± 0.02 | 53.38 ± 0.47 | 1.04 ± 0.01 |
| *L. crispatus* V342B | 0.17 ± 0.01 | 40.61 ± 0.93 | 4.15 ± 0.03 | 2.01 ± 0.03 | 55.20 ± 0.73 | 1.11 ± 0.09 |
| *L. iners* ATCC 55195 | 8.97 ± 0.03 | 216.90 ± 0.09 | 4.18 ± 0.02 | 2.88 ± 0.21 | 78.23 ± 5.53 | 1.14 ±0.09 |
| *L. reuteri* NCFB 2656^T^ | 11.12 ± 0.48 | 266.81 ± 0.26 | 4.42 ± 0.06 | 1.45 ± 0.01 | 40.27 ± 0.39 | 1.15 ± 0.04 |
| *L. fermentum* ATCC 11739^T^ | 0.17 ± 0.01 | 166.29 ± 0.48 | 2.83 ± 0.01 | 5.62 ± 0.08 | 151.26 ± 2.11 | 1.06 ± 0.04 |
| *L. jensenii* IM-USFQ | 11.39 ± 0.04 | 315.85 ± 1.35 | 2.66 ± 0.03 | 2.50 ± 0.01 | 68.30 ± 0.35 | 1.74 ± 0.07 |
| *Lactobacillus* sp. V156A | 9.02 ± 0.14 | 193.15 ± 0.74 | 3.60 ± 0.01 | 2.23 ± 0.06 | 61.11 ± 1.51 | 0.97 ± 0.05 |
| *Lactobacillus* sp*.* V174D | 8.64 ± 0.20 | 190.71 ± 0.82 | 2.82 ± 0.02 | 2.72 ± 0.04 | 74.21 ± 1.02 | 1.05 ± 0.04 |
| *Lactobacillus* sp*.* V221B | 7.61 ± 0.19 | 156.45 ± 0.57 | 2.78 ± 0.03 | 2.56 ± 0.07 | 69.82 ± 1.80 | 0.91 ± 0.08 |
| *Lactobacillus* sp. V280E | 9.70 ± 0.11 | 172.12 ± 0.39 | 3.70 ± 0.02 | 2.21 ± 0.03 | 60.42 ± 0.69 | 1.05 ± 0.06 |
| *Lactobacillus* sp. V310C | 14.14 ± 0.27 | 179.67 ± 2.49 | 5.01 ± 0.02 | 13.30 ± 0.11 | 356.03 ± 2.92 | 0.95 ± 0.05 |
| *Lactobacillus* sp. 102B | 0.17 ± 0.01 | 28.94 ± 0.12 | 2.06 ± 0.04 | 2.00 ± 0.08 | 54.97 ± 2.24 | 1.18 ± 0.04 |
| **Range** | 0.17 – 14.14 | 28.94 – 318.85 | 1.85 – 5.01 | 1.06 – 13.30 | 29.74 – 356.03 | 0.85 – 1.76 |
