# Supplementary material for: Cell-Free but potent: Antimicrobial potential of Lactobacillus supernatants against multidrug-resistant pathogens
Source: PLoS One. 2026 Aug 25;21(8):e0356730. doi: 10.1371/journal.pone.0356730 (PMC13505906; doi:10.1371/journal.pone.0356730)
Supplement: Supplementary file 6 [file pone.0356730.s006.docx]

**Supplementary Table S6.** Comparison of MIC and MBC or MFC values between ineffective CFS.

|  | **Cell-free supernatant (CFS)** | | | | |
| --- | --- | --- | --- | --- | --- |
|  | ***L. gasseri* V189D** | ***L. gasseri* V193B** | ***L. gasseri* V239B** | ***L. crispatus* V342B** | ***L. fermentum* ATCC 11739^T^** |
| ***Escherichia coli* ATCC 25922** | | | | | |
| MIC (mg/mL) | 60 | >80 | >80 | >80 | 40 |
| MBC (mg/mL) | 60 | >80 | >80 | >80 | 40 |
| Inhibition, SD (%)  Wilcoxon test *p*-value | 100 ±2.10  0.001015 | -6.78 ±4.37  0.3306 | -4.52 ±3.65  0.1139 | -8.35 ±4.15  0.00308 | 100 ±1.94  0.001756 |
| ***Escherichia coli* extended-spectrum beta-lactamases** | | | | | |
| MIC (mg/mL) | 60 | >80 | >80 | >80 | 40 |
| MBC (mg/mL) | 60 | >80 | >80 | >80 | 40 |
| Inhibition, SD (%)  Wilcoxon test *p*-value | 99.13 ±2.15  4.114E-05 | 16.76 ±4.48  0.008969 | -30.22 ±1.50  0.001739 | 13.44 ±2.50  0.002127 | 100 ±1.06  0.0004038 |
| ***Klebsiella pneumoniae* ATCC 700603** | | | | | |
| MIC (mg/mL) | 80 | >80 | >80 | 60 | 60 |
| MBC (mg/mL) | 80 | >80 | >80 | 60 | 60 |
| Inhibition, SD (%)  Wilcoxon test *p*-value | 100 ±0.61  0.0006258 | -27.99 ±4.03  0.0004038 | -21.89 ±3.42  0.0004095 | 100 ±0.45  0.000401 | 100 ±2.20  0.0004095 |
| ***Klebsiella pneumoniae* WT** | | | | | |
| MIC (mg/mL) | 80 | >80 | >80 | 60 | 80 |
| MBC (mg/mL) | 80 | >80 | >80 | 60 | 80 |
| Inhibition, SD (%)  Wilcoxon test *p*-value | 98.20 ±0.61  0.0005879 | -21.05 ±4.18  0.0004066 | -19.50 ±4.71  4.114E-05 | 100 ±2.64  4.114E-05 | 94.77 ±2.00  4.114E-05 |
| ***Klebsiella pneumoniae* carbapenemase-producing 609803** | | | | | |
| MIC (mg/mL) | 80 | >80 | >80 | 60 | 60 |
| MBC (mg/mL) | 80 | >80 | >80 | 60 | 60 |
| Inhibition, SD (%)  Wilcoxon test *p*-value | 99.88 ±3.20  0.0006306 | -46.21 ±4.05  8.227E-05 | -61.18 ±5.00  0.001711 | 88.34 ±3.84  0.0004066 | 97.43 ±1.59  0.0006306 |
| ***Pseudomonas aeruginosa* ATCC 27853** | | | | | |
| MIC (mg/mL) | 80 | >80 | >80 | 60 | >80 |
| MBC (mg/mL) | 80 | >80 | >80 | 60 | >80 |
| Inhibition, SD (%)  Wilcoxon test *p*-value | 96.30 ±2.69  4.114E-05 | -42.48 ±3.98  0.0004066 | 70.30 ±1.27  0.0004095 | 81.29 ±4.19  0.0004066 | 67.05 ±4.88  0.0004066 |
| ***Pseudomonas aeruginosa* P28** | | | | | |
| MIC (mg/mL) | 80 | >80 | >80 | 60 | 80 |
| MBC (mg/mL) | 80 | >80 | >80 | 80 | 80 |
| Inhibition, SD (%)  Wilcoxon test *p*-value | 96.47 ±1.63  8.227E-05 | 0.85 ±4.20  0.00757 | -41.59 ±3.97  0.000401 | 94.30 ±4.87  0.001024 | 98.07 ±3.18  0.001024 |
| ***Staphylococcus aureus* ATCC 25923** | | | | | |
| MIC (mg/mL) | 60 | >80 | >80 | 40 | 60 |
| MBC (mg/mL) | 60 | >80 | >80 | 40 | 60 |
| Inhibition, SD (%)  Wilcoxon test *p*-value | 96.36 ±2.21  8.227E-05 | 15.88 ±3.11  0.001486 | -7.54 ±4.84  0.08049 | 100 ±4.02  0.0001645 | 99.68 ±2.24  8.227E-05 |
| **Methicillin-resistant *Staphylococcus aureus* 333** | | | | | |
| MIC (mg/mL) | 60 | >80 | >80 | 60 | 60 |
| MBC (mg/mL) | 60 | >80 | >80 | 60 | 60 |
| Inhibition, SD (%)  Wilcoxon test *p*-value | 80.30 ±1.27  0.0004066 | 66.43 ±4.96  0.0004095 | -72.10 ±4.73  0.0004095 | 89.06 ±3.84  0.0006306 | 90.42 ±4.63  8.227E-05 |
| ***Enterococcus faecalis* ATCC 29212** | | | | | |
| MIC (mg/mL) | 40 | >80 | >80 | >80 | 80 |
| MBC (mg/mL) | 40 | >80 | >80 | >80 | 80 |
| Inhibition, SD (%)  Wilcoxon test *p*-value | 92.64 ±3.31  0.0004066 | -111.12 ±4.88  0.0004038 | -154.18 ±4.49  0.0004095 | 58.17 ±4.96  0.0004095 | 90.82 ±4.83  0.0003955 |
| **Vancomycin-resistant *Enterococcus faecalis* INSPI 032** | | | | | |
| MIC (mg/mL) | 80 | >80 | >80 | >80 | 40 |
| MBC (mg/mL) | 80 | >80 | >80 | >80 | 40 |
| Inhibition, SD (%)  Wilcoxon test *p*-value | 95.05±3.92  0.0006306 | 61.43 ±4.77  4.114E-05 | 21.35 ±4.52  4.114E-05 | 72.58 ±4.89  4.114E-05 | 100 ±3.81  0.0006258 |
| ***Enterococcus faecium* ATCC 27270** | | | | | |
| MIC (mg/mL) | >80 | >80 | >80 | 80 | 40 |
| MBC (mg/mL) | >80 | >80 | >80 | 80 | 40 |
| Inhibition, SD (%)  Wilcoxon test *p*-value | 58.26 ±4.85  0.0001554 | -395.05 ±3.88  0.0003955 | -356.97 ±4.25  0.0004095 | 86.13 ±4.18  8.227E-05 | 84.94 ±3.86  0.0003108 |
| ***Candida albicans* ATCC 10231** | | | | | |
| MIC (mg/mL) | 60 | >80 | >80 | 60 | >80 |
| MFC (mg/mL) | 80 | >80 | >80 | 60 | >80 |
| Inhibition, SD (%)  Wilcoxon test *p*-value | 100 ±2.36  0.001015 | -8.00 ±4.06  0.03595 | -26.64 ±4.93  0.0004066 | 94.27 ±4.79  0.001024 | 4.88 ±2.92  0.002626 |
| ***Candida tropicalis* WT** | | | | | |
| MIC (mg/mL) | >80 | >80 | >80 | >80 | 80 |
| MFC (mg/mL) | >80 | >80 | >80 | >80 | 80 |
| Inhibition, SD (%)  Wilcoxon test *p*-value | 0.12 ±4.93  0.03028 | -68.52 ±4.36  0.0004095 | -52.06 ±4.87  4.114E-05 | -33.24 ±3.13  0.0006306 | 84.82 ±2.96  0.0004066 |
| ***Candida tropicalis* INSPI 024** | | | | | |
| MIC (mg/mL) | >80 | >80 | >80 | >80 | 60 |
| MFC (mg/mL) | >80 | >80 | >80 | >80 | 80 |
| Inhibition, SD (%)  Wilcoxon test *p*-value | 0 ±4.97  0.1388 | -85.66 ±4.04  0.0006306 | -74.82 ±3.14  4.114E-05 | -61.74 ±3.64  0.0006306 | 85.29 ±1.45  0.0004095 |

The mean percentage inhibition and standard deviation (SD) were calculated using the mean values of the total number of assays (triplicate on three independent experiments). Negative inhibition percentages indicate that there was greater growth than the control without treatment. The Wilcoxon test was selected to evaluate the statistical differences between percentage inhibition without treatment vs percentage inhibition with CFS.
