# Supplementary material for: Cell-Free but potent: Antimicrobial potential of Lactobacillus supernatants against multidrug-resistant pathogens
Source: PLoS One. 2026 Aug 25;21(8):e0356730. doi: 10.1371/journal.pone.0356730 (PMC13505906; doi:10.1371/journal.pone.0356730)
Supplement: Supplementary file 7 [file pone.0356730.s007.docx]

**Supplementary Table S7.** Bacterial growth and viability in response to effective CFS and standard antimicrobials.

|  | **Cell-free supernatant (CFS)** | | | | | | | | | | **Antimicrobials** |
| --- | --- | --- | --- | --- | --- | --- | --- | --- | --- | --- | --- |
|  | ***L. plantarum* IM-USFQ** | ***L. plantarum* NCCB 46043^T^** | ***L. acidophilus* IM-USFQ** | ***L. acidophilus* ATCC 4356^T^** | ***L. gasseri* IM-USFQ** | ***L. gasseri* V254** | ***L. gasseri* V140B** | ***L. crispatus* V118C** | ***L. iners* ATCC 55195** | ***L. reuteri* NCFB 2656^T^** |  |
| ***Escherichia coli* ATCC 25922** | | | | | | | | | | | |
| OD_570nm_, SD  Wilcoxon test *p*-value | 0.123 ± 0.005  0.004998 | 0.068 ± 0.004  0.004998 | 0.063 ± 0.003  0.004998 | 0.063 ± 0.005  0.004998 | 0.061 ± 0.001  0.004698 | 0.063 ± 0.004  0.002165 | 0.068 ± 0.001  0.004698 | 0.081 ± 0.004  0.002165 | 0.074 ± 0.004  0.004772 | 0.062 ± 0.002  0.004772 | 0.055 ± 0.002^CIP^  0.0003954938 |
| log (CFU/mL), SD  Wilcoxon test *p*-value | 4.436 ± 0.121  2.592E-07 | 2.586 ± 0.125  2.794E-07 | 2.000 ± 0.001  4.584E-08 | 3.973 ± 0.047  4.778E-07 | 3.851 ± 0.022  3.101E-07 | 3.259 ± 0.105  3.172E-07 | 3.174 ± 0.099  3.106E-07 | 3.670 ± 0.075  3.139E-07 | 2.000 ± 0.001  4.584E-08 | 2.379 ± 0.090  1.955E-07 | 4.460 ± 0.091^CIP^  3.122606E-07 |
| ***Escherichia coli* extended-spectrum beta-lactamases** | | | | | | | | | | | |
| OD_570nm_, SD  Wilcoxon test *p*-value | 0.097 ± 0.009  0.002165 | 0.063 ± 0.004  0.002165 | 0.063 ± 0.001  0.004267 | 0.089 ± 0.010  0.002165 | 0.065 ± 0.008  0.004998 | 0.067 ± 0.005  0.004998 | 0.067 ± 0.003  0.004922 | 0.065 ± 0.007  0.004998 | 0.069 ± 0.006  0.002165 | 0.065 ± 0.003  0.002165 | 0.056 ± 0.001^CIP^  0.0003712137 |
| log (CFU/mL), SD  Wilcoxon test *p*-value | 3.810 ± 0.015  2.999E-07 | 3.159 ± 0.093  3.074E-07 | 3.228 ± 0.064  3.036E-07 | 3.319 ± 0.044  3.026E-07 | 3.034 ± 0.053  2.824E-07 | 3.408 ± 0.067  3.095E-07 | 4.257 ± 0.025  3.194E-07 | 3.115 ± 0.086  3.085E-07 | 3.375 ± 0.072  3.09E-07 | 2.000 ± 0.001  4.594E-08 | 4.236 ±0.178^CIP^  3.133543E-07 |
| ***Klebsiella pneumoniae* ATCC 700603** | | | | | | | | | | | |
| OD_570nm_, SD  Wilcoxon test *p*-value | 0.067 ± 0.004  0.002165 | 0.056 ± 0.003  0.004922 | 0.072 ± 0.009  0.002165 | 0.059 ± 0.009  0.004772 | 0.074 ± 0.009  0.002165 | 0.064 ± 0.010  0.002165 | 0.092 ± 0.004  0.004998 | 0.077 ± 0.008  0.004998 | 0.077 ± 0.004  0.004998 | 0.059 ± 0.002  0.004998 | 0.063 ± 0.008^CIP^  0.0004066393 |
| log (CFU/mL), SD  Wilcoxon test *p*-value | 5.701 ± 0.028  1.023E-06 | 3.991 ± 0.019  7.205E-07 | 2.000 ± 0.001  4.699E-08 | 5.824 ± 0.018  7.163E-07 | 5.886 ± 0.021  3.128E-07 | 6.382 ± 0.054  7.163E-07 | 2.067 ± 0.129  1.361E-07 | 5.372 ± 0.079  3.079E-07 | 5.726 ± 0.045  3.189E-07 | 3.560 ± 0.039  7.177E-07 | 4.436 ± 0.159^CIP^  2.715907E-07 |
| ***Klebsiella pneumoniae* WT** | | | | | | | | | | | |
| OD_570nm_, SD  Wilcoxon test *p*-value | 0.063 ± 0.003  0.002165 | 0.061 ± 0.004  0.004998 | 0.116 ± 0.010  0.002165 | 0.063 ± 0.006  0.002165 | 0.155 ± 0.003  0.004998 | 0.060 ± 0.003  0.002165 | 0.116 ± 0.008  0.002165 | 0.112 ± 0.008  0.004998 | 0.125 ± 0.005  0.002165 | 0.061 ± 0.007  0.004998 | 0.056 ± 0.005^CIP^  0.0004094601 |
| log (CFU/mL), SD  Wilcoxon test *p*-value | 3.903 ± 0.046  3.156E-07 | 3.907 ± 0.030  3.167E-07 | 2.000 ± 0.001  4.689E-08 | 6.133 ± 0.015  3.145E-07 | 5.146 ± 0.095  3.117E-07 | 5.849 ± 0.076  4.752E-07 | 6.599 ± 0.074  3.2E-07 | 3.703 ± 0.068  3.189E-07 | 5.565 ± 0.031  3.134E-07 | 5.120 ± 0.070  3.036E-07 | 5.488 ± 0.065^CIP^  3.188721E-07 |
| ***Klebsiella pneumoniae* carbapenemase-producing 609803** | | | | | | | | | | | |
| OD_570nm_, SD  Wilcoxon test *p*-value | 0.064 ± 0.001  0.004772 | 0.057 ± 0.002  0.002165 | 0.075 ± 0.010  0.002165 | 0.057 ± 0.001  0.004772 | 0.075 ± 0.010  0.002165 | 0.065 ± 0.008  0.002165 | 0.092 ± 0.004  0.004998 | 0.077 ± 0.010  0.002165 | 0.073 ± 0.010  0.002165 | 0.068 ± 0.009  0.002165 | 0.070 ± 0.009^CIP^  0.0003845673 |
| log (CFU/mL), SD  Wilcoxon test *p*-value | 3.711 ± 0.040  3.134E-07 | 3.865 ± 0.015  3.095E-07 | 2.000 ± 0.001  4.594E-08 | 4.051 ± 0.019  7.065E-07 | 3.223 ± 0.066  3.058E-07 | 3.753 ± 0.058  4.708E-07 | 3.454 ± 0.094  3.15E-07 | 4.024 ± 0.064  3.128E-07 | 2.717 ± 0.138  2.916E-07 | 2.677 ± 0.126  2.523E-07 | 5.928 ± 0.027^CIP^  3.139024E-07 |
| ***Pseudomonas aeruginosa* ATCC 27853** | | | | | | | | | | | |
| OD_570nm_, SD  Wilcoxon test *p*-value | 0.080 ± 0.008  0.002165 | 0.062 ± 0.004  0.004922 | 0.064 ± 0.002  0.004847 | 0.059 ± 0.004  0.004698 | 0.060 ± 0.001  0.004624 | 0.062 ± 0.002  0.004998 | 0.085 ± 0.002  0.004847 | 0.062 ± 0.001  0.004624 | 0.063 ± 0.002  0.004922 | 0.062 ± 0.002  0.004624 | 0.091 ± 0.006^CIP^  0.000401039 |
| log (CFU/mL), SD  Wilcoxon test *p*-value | 3.471 ± 0.031  3.085E-07 | 5.731 ± 0.092  3.194E-07 | 4.075 ± 0.020  3.052E-07 | 5.677 ± 0.085  3.183E-07 | 3.934 ± 0.037  3.074E-07 | 4.034 ± 0.014  3.123E-07 | 3.141 ± 0.058  2.87E-07 | 6.040 ± 0.029  3.063E-07 | 3.143 ± 0.058  2.921E-07 | 3.983 ± 0.025  3.189E-07 | 5.565 ± 0.025^CIP^  2.931247E-07 |
| ***Pseudomonas aeruginosa* P28** | | | | | | | | | | | |
| OD_570nm_, SD  Wilcoxon test *p*-value | 0.074 ± 0.005  0.004998 | 0.062 ± 0.003  0.004922 | 0.062 ± 0.001  0.004698 | 0.066 ± 0.001  0.004998 | 0.059 ± 0.001  0.004772 | 0.067 ± 0.004  0.004772 | 0.066 ± 0.003  0.002165 | 0.060 ± 0.001  0.004267 | 0.062 ± 0.004  0.004998 | 0.064 ± 0.002  0.004998 | 0.059 ± 0.004^CIP^  0.000401039 |
| log (CFU/mL), SD  Wilcoxon test *p*-value | 4.452 ± 0.163  2.668E-07 | 4.363 ± 0.126  3.434E-07 | 4.006 ± 0.034  3.063E-07 | 4.773 ± 0.144  3.074E-07 | 3.940 ± 0.044  3.079E-07 | 3.498 ± 0.033  3.042E-07 | 3.447 ± 0.058  3.042E-07 | 5.978 ± 0.039  3.063E-07 | 3.128 ± 0.060  2.921E-07 | 4.332 ± 0.188  4.009E-07 | 3.691 ± 0.171^CIP^  2.920917E-07 |
| ***Staphylococcus aureus* ATCC 25923** | | | | | | | | | | | |
| OD_570nm_, SD  Wilcoxon test *p*-value | 0.082 ± 0.010  0.002165 | 0.108 ± 0.010  0.004998 | 0.066 ± 0.001  0.004698 | 0.067 ± 0.003  0.002165 | 0.063 ± 0.002  0.004698 | 0.065 ± 0.002  0.004998 | 0.091± 0.003  0.002165 | 0.079 ± 0.007  0.002165 | 0.063 ± 0.001  0.004337 | 0.067 ± 0.002  0.004772 | 0.062 ± 0.008^CIP^  0.0004066393 |
| log (CFU/mL), SD  Wilcoxon test *p*-value | 5.960 ± 0.039  3.2E-07 | 6.104 ± 0.015  3.178E-07 | 5.481 ± 0.113  3.079E-07 | 5.927 ± 0.046  4.787E-07 | 5.389 ± 0.085  3.052E-07 | 6.068 ± 0.035  3.205E-07 | 6.420 ± 0.016  3.09E-07 | 5.536 ± 0.044  3.015E-07 | 5.482 ± 0.075  3.074E-07 | 6.038 ± 0.054  3.2E-07 | 5.143 ± 0.037^CIP^  3.095406E-07 |
| **Methicillin-resistant *Staphylococcus aureus* 333** | | | | | | | | | | | |
| OD_570nm_, SD  Wilcoxon test *p*-value | 0.178 ± 0.003  0.004922 | 0.108 ± 0.010  0.004922 | 0.070 ± 0.006  0.004922 | 0.113 ± 0.008  0.004998 | 0.069 ± 0.008  0.004998 | 0.065 ± 0.001  0.004772 | 0.090 ± 0.005  0.004998 | 0.097 ± 0.003  0.004922 | 0.066 ± 0.003  0.004998 | 0.069 ± 0.003  0.004922 | 0.087 ± 0.009^CIP^  0.0003927418 |
| log (CFU/mL), SD  Wilcoxon test *p*-value | 5.867 ± 0.091  4.761E-07 | 6.074 ± 0.028  7.332E-07 | 5.572 ± 0.107  7.233E-07 | 5.865 ± 0.029  4.726E-07 | 5.643 ± 0.149  1.051E-06 | 6.166 ± 0.022  3.205E-07 | 5.848 ± 0.053  1.049E-06 | 6.190 ± 0.010  7.219E-07 | 5.738 ± 0.073  7.318E-07 | 6.189 ± 0.014  3.178E-07 | 5.293 ± 0.086^CIP^  3.144513E-07 |
| ***Enterococcus faecalis* ATCC 29212** | | | | | | | | | | | |
| OD_570nm_, SD  Wilcoxon test *p*-value | 0.149 ± 0.002  0.002165 | 0.098 ± 0.009  0.002165 | 0.064 ± 0.002  0.004998 | 0.102 ± 0.008  0.004998 | 0.062 ± 0.004  0.004772 | 0.063 ± 0.001  0.004847 | 0.070 ± 0.007  0.007969 | 0.094 ± 0.008  0.004998 | 0.071 ± 0.006  0.004329 | 0.065 ± 0.001  0.004847 | 0.050 ± 0.001^AMP^  0.0003738576 |
| log (CFU/mL), SD  Wilcoxon test *p*-value | 6.048 ± 0.025  3.156E-07 | 6.398 ± 0.050  3.079E-07 | 3.794 ± 0.073  3.161E-07 | 6.424 ± 0.046  3.079E-07 | 5.916 ± 0.061  3.15E-07 | 6.500 ± 0.049  3.117E-07 | 6.351 ± 0.128  3.145E-07 | 6.691 ± 0.049  3.15E-07 | 6.160 ± 0.018  3.156E-07 | 6.388 ± 0.045  3.085E-07 | 3.077 ± 0.152^AMP^  1.108114E-07 |
| **Vancomycin-resistant *Enterococcus faecalis* INSPI 032** | | | | | | | | | | | |
| OD_570nm_, SD  Wilcoxon test *p*-value | 0.142 ± 0.008  0.004329 | 0.066 ± 0.004  0.004329 | 0.077 ± 0.005  0.002165 | 0.122 ± 0.006  0.004329 | 0.081 ± 0.007  0.002165 | 0.062 ± 0.002  0.004922 | 0.094 ± 0.007  0.002165 | 0.090 ± 0.008  0.004998 | 0.089 ± 0.008  0.004998 | 0.071 ± 0.006  0.004998 | 0.058 ± 0.005^AMP^  0.0004038323 |
| log (CFU/mL), SD  Wilcoxon test *p*-value | 5.985 ± 0.052  3.139E-07 | 5.871 ± 0.050  3.161E-07 | 5.539 ± 0.034  3.079E-07 | 5.852 ± 0.028  3.167E-07 | 5.921 ± 0.078  3.172E-07 | 6.093 ± 0.051  3.189E-07 | 5.328 ± 0.076  4.656E-07 | 5.661 ± 0.066  4.7E-07 | 5.916 ± 0.038  3.15E-07 | 5.710 ± 0.103  3.167E-07 | 4.752 ± 0.042^AMP^  3.11715E-07 |
| ***Enterococcus faecium* ATCC 27270** | | | | | | | | | | | |
| OD_570nm_, SD  Wilcoxon test *p*-value | 0.156 ± 0.007  0.004998 | 0.070 ± 0.009  0.004998 | 0.063 ± 0.003  0.004922 | 0.143 ± 0.006  0.004998 | 0.063 ± 0.004  0.004998 | 0.065 ± 0.001  0.004267 | 0.074 ± 0.010  0.004998 | 0.088 ± 0.009  0.004998 | 0.063 ± 0.005  0.004922 | 0.068 ± 0.002  0.004922 | 0.053 ± 0.003^AMP^  0.0004038323 |
| log (CFU/mL), SD  Wilcoxon test *p*-value | 5.816 ± 0.021  4.743E-07 | 5.802 ± 0.025  3.161E-07 | 4.198 ± 0.012  3.134E-07 | 5.804 ± 0.019  3.156E-07 | 6.007 ± 0.022  3.15E-07 | 5.842 ± 0.037  3.194E-07 | 5.398 ± 0.086  3.178E-07 | 6.022 ± 0.017  3.178E-07 | 3.862 ± 0.033  3.161E-07 | 5.829 ± 0.026  3.112E-07 | 4.503 ± 0.067^AMP^  3.172081E-07 |
| ***Candida albicans* ATCC 10231** | | | | | | | | | | | |
| OD_570nm_, SD  Wilcoxon test *p*-value | 0.176 ± 0.003  0.002165 | 0.101 ± 0.007  0.002165 | 0.126 ± 0.015  0.002165 | 0.111 ± 0.010  0.002165 | 0.146 ± 0.010  0.004998 | 0.066 ± 0.003  0.004922 | 0.092 ± 0.007  0.004998 | 0.145 ± 0.010  0.002165 | 0.130 ± 0.008  0.004998 | 0.067 ± 0.003  0.004922 | 0.054 ± 0.005^FCZ^  0.0004094601 |
| log (CFU/mL), SD  Wilcoxon test *p*-value | 3.983 ± 0.028  4.743E-07 | 3.231 ± 0.088  4.631E-07 | 5.189 ± 0.076  3.085E-07 | 3.762 ± 0.063  4.734E-07 | 4.760 ± 0.133  2.994E-07 | 3.507 ± 0.045  4.674E-07 | 6.474 ± 0.094  3.156E-07 | 5.850 ± 0.032  3.172E-07 | 5.454 ± 0.083  3.145E-07 | 3.172 ± 0.076  4.613E-07 | 4.697 ± 0.058^FCZ^  3.155515E-07 |
| ***Candida tropicalis* WT** | | | | | | | | | | | |
| OD_570nm_, SD  Wilcoxon test *p*-value | 0.259 ± 0.006  0.002165 | 0.191 ± 0.005  0.002165 | 0.224 ± 0.008  0.004998 | 0.227 ± 0.009  0.002165 | 0.224 ± 0.010  0.002165 | 0.198 ± 0.009  0.002165 | 0.246 ± 0.009  0.002165 | 0.227 ± 0.005  0.002165 | 0.244 ± 0.010  0.002165 | 0.244 ± 0.008  0.004998 | 0.057 ± 0.005^FCZ^  0.0004094601 |
| log (CFU/mL), SD  Wilcoxon test *p*-value | 6.170 ± 0.011  4.613E-07 | 6.137 ± 0.012  4.691E-07 | 6.820 ± 0.030  3.183E-07 | 6.199 ± 0.015  4.7E-07 | 6.884 ± 0.019  4.691E-07 | 6.193 ± 0.022  4.708E-07 | 6.990 ± 0.015  3.128E-07 | 6.959 ± 0.021  3.172E-07 | 6.908 ± 0.033  3.156E-07 | 6.176 ± 0.012  4.665E-07 | 4.480 ± 0.086^FCZ^  3.15001E-07 |
| ***Candida tropicalis* INSPI 024** | | | | | | | | | | | |
| OD_570nm_, SD  Wilcoxon test *p*-value | 0.266 ± 0.010  0.004998 | 0.272 ± 0.008  0.004998 | 0.249 ± 0.009  0.004998 | 0.257 ± 0.007  0.002165 | 0.258 ± 0.009  0.004998 | 0.204 ± 0.010  0.002165 | 0.273 ± 0.010  0.004998 | 0.248 ± 0.009  0.002165 | 0.271 ± 0.009  0.002165 | 0.249 ± 0.005  0.004922 | 0.056 ± 0.003^FCZ^  0.0003982596 |
| log (CFU/mL), SD  Wilcoxon test *p*-value | 6.291 ± 0.005  3.004E-07 | 6.206 ± 0.013  3.047E-07 | 6.867 ± 0.036  3.139E-07 | 6.144 ± 0.021  3.085E-07 | 6.923 ± 0.027  3.134E-07 | 4.275 ± 0.133  3.068E-07 | 7.031 ± 0.019  3.145E-07 | 6.964 ± 0.025  3.172E-07 | 7.000 ± 0.011  3.02E-07 | 6.229 ± 0.013  3.095E-07 | 4.545 ± 0.043^FCZ^  3.144513E-07 |

CIP: ciprofloxacin; AMP: ampicillin; FCZ: fluconazole. The mean optical density (OD_570nm_) and log (CFU/mL), and standard deviation were calculated using the mean values of the total number of assays (triplicate on three independent experiments). The Wilcoxon test was selected to evaluate the statistical differences between percentage inhibition without treatment vs percentage inhibition with treatment (different CFS and antimicrobial control).
