# Supplementary material for: Cell-Free but potent: Antimicrobial potential of Lactobacillus supernatants against multidrug-resistant pathogens
Source: PLoS One. 2026 Aug 25;21(8):e0356730. doi: 10.1371/journal.pone.0356730 (PMC13505906; doi:10.1371/journal.pone.0356730)
Supplement: Supplementary file 9 [file pone.0356730.s009.docx]

**Supplementary Table S9.** Quantitative fluorescence microscopy data.

| **Pathogenic strain** | **Parameters** | **Control without CFS** | **Antimicrobial control** | **With CFS** | **Wilcoxon test *p*-value** | | |
| --- | --- | --- | --- | --- | --- | --- | --- |
|  |  |  |  |  | **Control without CFS vs Antimicrobial control** | **Control without CFS vs With CFS** | **Antimicrobial control vs With CFS** |
| *E. coli* ATCC 25922 | Total cell count, (cells/frame) ± SD | 734.93 ± 529.93 | 1.73 ± 1.18 | 3.39 ± 3.11 | 4.560856E-20 | 3.424663E-18 | 0.0003054932 |
|  | Total cell count, (cells/cm^2^) ± SD | 5.66E+06 ± 4.08E+06 | 1.33E+04 ± 9.12E+03 | 2.61E+04 ± 3.09E+03 | 4.55998E-20 | 3.423961E-18 | 0.0003054932 |
|  | Live cells, (%) ± SEM | 98.78 ± 0.16 | 10.72 ± 3.25 | 19.30 ± 4.22 | 7.254531E-18 | 3.027833E-13 | 0.1184182 |
| *E. coli* extended-spectrum beta-lactamases | Total cell count, (cells/frame) ± SD | 433.96 ± 234.97 | 3.2 ± 1.9 | 2.67 ± 3.09 | 1.353017E-20 | 5.058734E-19 | 0.009944932 |
|  | Total cell count, (cells/cm^2^) ± SD | 3.34E+06 ± 2.34E+05 | 2.46E+04 ± 1.87E+03 | 2.06E+04 ± 3.07E+03 | 1.353017E-20 | 1.05069E-18 | 0.009944932 |
|  | Live cells, (%) ± SEM | 96.64 ± 0.62 | 14.62 ± 3.12 | 22.26 ± 3.92 | 4.705643E-19 | 5.881898E-16 | 0.1927632 |
| *K.pneumoniae* ATCC 700603 | Total cell count, (cells/frame) ± SD | 1620.27 ± 922.53 | 2.47 ± 2.29 | 2.03 ± 1.21 | 1.99206E-21 | 1.927493E-21 | 0.7794379 |
|  | Total cell count, (cells/cm^2^) ± SD | 1.25E+07 ± 7.10E+06 | 1.90E+04 ± 1.76E+04 | 1.57E+04 ± 1.20E+03 | 1.989822E-21 | 1.925324E-21 | 0.7794379 |
|  | Live cells, (%) ± SEM | 95.53 ± 0.51 | 17.25 ± 2.69 | 24.46 ± 4.80 | 1.159351E-21 | 1.541295E-12 | 0.8835652 |
| *K. pneumoniae* WT | Total cell count, (cells/frame) ± SD | 608.57 ± 680.07 | 3.19 ± 2.07 | 3.34 ± 2.95 | 7.958238E-20 | 4.800054E-17 | 0.4862169 |
|  | Total cell count, (cells/cm^2^) ± SD | 4.69E+06 ± 5.24E+06 | 2.46E+04 ± 1.59E+04 | 2.57E+04 ± 2.27E+04 | 7.955372E-20 | 4.798803E-17 | 0.4862169 |
|  | Live cells, (%) ± SEM | 99.76 ± 0.07 | 20.77 ± 2.67 | 19.97 ± 4.10 | 1.372902E-20 | 1.054513E-15 | 0.1899389 |
| *K. pneumoniae* carbapenemase-producing 609803 | Total cell count, (cells/frame) ± SD | 621.17 ± 366.95 | 5.74 ± 2.37 | 4.06 ± 5.12 | 6.671027E-21 | 3.319534E-15 | 8.560333E-06 |
|  | Total cell count, (cells/cm^2^) ± SD | 4.78E+06 ± 2.83E+06 | 4.42E+04 ± 1.83E+04 | 3.13E+04 ± 3.95E+04 | 6.667767E-21 | 5.183773E-12 | 8.560333E-06 |
|  | Live cells, (%) ± SEM | 96.21 ± 0.38 | 29.69 ± 2.89 | 30.50 ± 4.87 | 6.896313E-21 | 1.425954E-06 | 0.2270306 |
| *P. aeruginosa* ATCC 27853 | Total cell count, (cells/frame) ± SD | 213.43 ± 249.79 | 2.65 ± 1.88 | 7.49 ± 9.64 | 2.409944E-19 | 4.843247E-15 | 5.533651E-05 |
|  | Total cell count, (cells/cm^2^) ± SD | 1.64E+06 ± 1.92E+06 | 2.04E+04 ± 1.45E+04 | 5.77E+04 ± 7.42E+04 | 8.07486E-19 | 1.091807E-14 | 5.533651E-05 |
|  | Live cells, (%) ± SEM | 98.73 ± 0.40 | 39.23 ± 4.87 | 35.24 ± 4.79 | 1.110684E-12 | 2.85191E-12 | 0.7692961 |
| *P. aeruginosa* P28 | Total cell count, (cells/frame) ± SD | 453.98 ± 191.49 | 2.6 ± 1.45 | 2.77 ± 2.24 | 5.447073E-20 | 1.0504E-17 | 0.5987482 |
|  | Total cell count, (cells/cm^2^) ± SD | 3.50E+06 ± 1.47E+06 | 2.00E+04 ± 1.11E+04 | 2.14E+04 ± 1.72E+04 | 3.934244E-20 | 1.670958E-20 | 0.5987482 |
|  | Live cells, (%) ± SEM | 94.64 ± 0.72 | 38.44 ± 4.63 | 37.34 ± 5.45 | 5.69156E-12 | 4.71551E-07 | 0.6753393 |
| *S. aureus* ATCC 25923 | Total cell count, (cells/frame) ± SD | 1018.25 ± 691.28 | 3.31 ± 1.61 | 3.71 ± 2.21 | 2.899329E-21 | 3.035124E-21 | 0.4910451 |
|  | Total cell count, (cells/cm^2^) ± SD | 7.84E+06 ± 5.32E+06 | 2.55E+04 ± 1.24E+04 | 2.86E+04 ± 1.70E+04 | 6.376355E-21 | 3.033689E-21 | 0.4910451 |
|  | Live cells, (%) ± SEM | 97.82 ± 0.56 | 44.50 ± 4.79 | 48.06 ± 4.19 | 1.241073E-10 | 4.831694E-11 | 0.664788 |
| Methicillin-resistant *S. aureus* 333 | Total cell count, (cells/frame) ± SD | 689.30 ± 111.83 | 2.94 ± 2.10 | 3.48 ± 2.66 | 2.959245E-10 | 2.576334E-11 | 0.6982726 |
|  | Total cell count, (cells/cm^2^) ± SD | 5.31E+06 ± 8.61E+06 | 2.27E+04 ± 1.62E+04 | 2.68E+04 ± 2.05E+04 | 2.957227E-10 | 2.5746E-11 | 0.6982726 |
|  | Live cells, (%) ± SEM | 98.82 ± 0.37 | 42.72 ± 4.14 | 39.23 ± 4.85 | 1.497675E-05 | 8.946427E-07 | 0.8563612 |
| *E. faecalis* ATCC 29212 | Total cell count, (cells/frame) ± SD | 848.89 ± 341.53 | 1.94 ± 1.39 | 2.17 ± 1.34 | 4.044416E-16 | 1.457625E-12 | 0.3259149 |
|  | Total cell count, (cells/cm^2^) ± SD | 6.54E+06 ± 2.63E+06 | 1.50E+04 ± 1.07E+04 | 1.67E+04 ± 1.03E+04 | 4.044416E-16 | 1.457625E-12 | 0.3259149 |
|  | Live cells, (%) ± SEM | 96.78 ± 0.57 | 38.94 ± 5.48 | 38.19 ± 5.17 | 1.988719E-06 | 3.35478E-06 | 0.9807469 |
| Vancomycin-resistant *E. faecalis* INSPI 032 | Total cell count, (cells/frame) ± SD | 1255.19 ± 914.15 | 2.78 ± 2.07 | 4.00 ± 3.29 | 9.906234E-18 | 1.425554E-14 | 0.08487443 |
|  | Total cell count, (cells/cm^2^) ± SD | 9.66E+06 ± 7.04E+06 | 2.14E+04 ± 1.60E+04 | 3.08E+04 ± 2.53E+04 | 9.906234E-18 | 1.425554E-14 | 0.08487443 |
|  | Live cells, (%) ± SEM | 91.11 ± 0.71 | 39.40 ± 5.13 | 38.88 ± 5.64 | 7.61913E-06 | 0.0001254032 | 0.8022882 |
| *E. faecium* ATCC 27270 | Total cell count, (cells/frame) ± SD | 1036.68 ± 1251.18 | 1.74 ± 1.10 | 1.76 ± 1.30 | 2.48368E-18 | 4.242131E-14 | 0.7254815 |
|  | Total cell count, (cells/cm^2^) ± SD | 7.98E+06 ± 9.63E+06 | 1.34E+04 ± 8.50E+03 | 1.35E+04 ± 1.00E+04 | 2.48368E-18 | 4.242131E-14 | 0.7254815 |
|  | Live cells, (%) ± SEM | 83.64 ± 1.32 | 40.55 ± 5.59 | 38.18 ± 5.90 | 0.0001719274 | 0.003683077 | 0.7896075 |
| *C. albicans* ATCC 10231 | Total cell count, (cells/frame) ± SD | 99.31 ± 99.59 | 7.35 ± 7.80 | 3.15 ± 3.02 | 1.722624E-09 | 2.439804E-12 | 0.0009686517 |
|  | Total cell count, (cells/cm^2^) ± SD | 7.65E+05 ± 7.67E+05 | 5.66E+04 ± 6.01E+04 | 2.42E+04 ± 2.32E+04 | 1.722624E-09 | 2.439804E-12 | 0.0009686517 |
|  | Live cells, (%) ± SEM | 97.99 ± 0.39 | 15.12 ± 2.10 | 22.35 ± 3.49 | 1.240723E-12 | 2.891585E-12 | 0.5786887 |
| *C. tropicalis* WT | Total cell count, (cells/frame) ± SD | 60.6 ± 64.54 | 2.58 ± 1.67 | 2.49 ± 1.51 | 2.235913E-14 | 3.359261E-20 | 0.8216422 |
|  | Total cell count, (cells/cm^2^) ± SD | 4.67E+05 ± 4.97E+05 | 1.99E+04 ± 1.28E+04 | 1.92E+04 ± 1.17E+04 | 2.235913E-14 | 3.359261E-20 | 0.8216422 |
|  | Live cells, (%) ± SEM | 92.53 ± 1.06 | 15.23 ± 3.05 | 23.12 ± 4.01 | 9.626704E-14 | 7.42253E-16 | 0.2984915 |
| *C. tropicalis* INSPI 024 | Total cell count, (cells/frame) ± SD | 100.50 ± 77.80 | 2.07 ± 1.13 | 3.68 ± 4.23 | 1.459837E-17 | 1.279533E-20 | 0.06062359 |
|  | Total cell count, (cells/cm^2^) ± SD | 7.74E+05 ± 5.99E+05 | 1.60E+04 ± 8.68E+03 | 2.84E+04 ± 3.26E+04 | 1.459837E-17 | 1.279533E-20 | 0.06062359 |
|  | Live cells, (%) ± SEM | 90.76 ± 1.04 | 21.75 ± 4.52 | 27.72 ± 4.59 | 2.939533E-11 | 6.218489E-12 | 0.200838 |

The mean total cell counts and percentage of live cells with standard deviation (SD) were calculated using the mean values of the total number of assays (triplicate on three independent experiments). The Wilcoxon test was selected to evaluate the statistical differences between data without treatment vs data with treatment (different CFS and antimicrobial control).
