# Supplementary material for: Cell-Free but potent: Antimicrobial potential of Lactobacillus supernatants against multidrug-resistant pathogens
Source: PLoS One. 2026 Aug 25;21(8):e0356730. doi: 10.1371/journal.pone.0356730 (PMC13505906; doi:10.1371/journal.pone.0356730)
Supplement: Supplementary file 10 [file pone.0356730.s010.docx]

**Supplementary Table S10.** Effects of pH neutralization on the antimicrobial activity of CFS.

|  | **Cell-free supernatant (CFS)** | | | | | | | | | |
| --- | --- | --- | --- | --- | --- | --- | --- | --- | --- | --- |
|  | ***L. plantarum* IM-USFQ** | ***L. plantarum* NCCB 46043^T^** | ***L. acidophilus* IM-USFQ** | ***L. acidophilus* ATCC 4356^T^** | ***L. gasseri* IM-USFQ** | ***L. gasseri* V254** | ***L. gasseri* V140B** | ***L. crispatus* V118C** | ***L. iners* ATCC 55195** | ***L. reuteri* NCFB 2656^T^** |
| pH | 4.32 | 4.23 | 4.31 | 4.21 | 4.99 | 4.17 | 4.55 | 4.30 | 4.25 | 4.26 |
| ***Escherichia coli* ATCC 25922** | | | | | | | | | | |
| Inhibition at acid pH, SD (%) | 100 ± 0.96 | 100 ± 0.89 | 100 ± 1.19 | 100 ± 3.85 | 99.00 ± 0.96 | 100 ± 3.44 | 100 ± 1.32 | 100 ± 0.57 | 100 ± 0.78 | 100 ± 1.17 |
| Inhibition at neutral pH, SD (%) | 24.53 ± 4.29 | 3.96 ± 5.31 | 36.35 ± 4.84 | 28.60 ± 3.82 | 35.88 ± 3.78 | 7.17 ± 5.34 | 34.84 ± 3.38 | 22.84 ± 5.19 | -7.35 ± 4.23 | 15.50 ± 3.67 |
| Wilcoxon test *p*-value | 0.0006306361 | 0.0006306361 | 8.227067E-05 | 0.0004094601 | 0.0006306361 | 8.227067E-05 | 0.0004094601 | 0.0006209699 | 4.113534E-05 | 0.0004094601 |
| ***Escherichia coli* extended-spectrum beta-lactamases** | | | | | | | | | | |
| Inhibition at acid pH, SD (%) | 100 ± 1.47 | 100 ± 0.80 | 100 ± 1.07 | 100 ± 1.31 | 100 ± 1.31 | 100 ± 0.84 | 100 ± 0.75 | 100 ± 0.65 | 100 ± 0.78 | 100 ± 1.77 |
| Inhibition at neutral pH, SD (%) | 14.03 ± 4.00 | 17.34 ± 2.01 | 17.36 ± 3.66 | 34.08 ± 1.74 | 25.67 ± 4.24 | 18.95 ± 4.05 | 30.54 ± 3.64 | 35.57 ± 4.14 | 21.93 ± 4.84 | 15.57 ± 4.09 |
| Wilcoxon test *p*-value | 0.0006306361 | 0.0009148479 | 0.0006306361 | 0.0006306361 | 8.227067E-05 | 0.0004094601 | 0.0006257901 | 0.0001554002 | 0.0009309723 | 0.0006257901 |
| ***Klebsiella pneumoniae* ATCC 700603** | | | | | | | | | | |
| Inhibition at acid pH, SD (%) | 95.63 ± 1.39 | 98.77 ± 4.47 | 99.84 ± 0.86 | 100 ± 2.75 | 99.31 ± 2.64 | 100 ± 1.13 | 99.67 ± 1.31 | 98.73 ± 4.02 | 98.66 ± 2.45 | 100 ± 1.06 |
| Inhibition at neutral pH, SD (%) | 0.30 ± 1.48 | 2.34 ± 3.41 | 2.67 ± 3.90 | 2.54 ± 3.92 | 4.06 ± 2.94 | 2.58 ± 3.21 | 3.43 ± 2.96 | 2.15 ± 4.11 | 2.93 ± 2.72 | 2.70 ± 3.97 |
| Wilcoxon test *p*-value | 4.113534E-05 | 0.0004094601 | 0.0004094601 | 0.0004094601 | 0.0004038323 | 0.0004094601 | 0.0004066393 | 4.113534E-05 | 4.113534E-05 | 0.0004094601 |
| ***Klebsiella pneumoniae* WT** | | | | | | | | | | |
| Inhibition at acid pH, SD (%) | 100 ± 0.85 | 100 ± 3.44 | 97.75 ± 2.20 | 100 ± 1.07 | 100 ± 3.67 | 100 ± 4.58 | 92.44 ± 3.77 | 100 ± 2.46 | 100 ± 1.89 | 100 ± 0.97 |
| Inhibition at neutral pH, SD (%) | -3.05 ± 4.16 | 11.77 ± 3.19 | -2.82 ± 4.83 | 25.31 ± 5.11 | 7.92 ± 5.31 | 17.08 ± 4.21 | 0.29 ± 3.87 | 10.63 ± 2.90 | 0.62 ± 2.72 | 3.89 ± 4.56 |
| Wilcoxon test *p*-value | 0.0004066393 | 0.0004094601 | 0.0004066393 | 0.0004066393 | 0.0004094601 | 0.0004066393 | 0.0004066393 | 0.0004066393 | 0.0004094601 | 0.0004094601 |
| ***Klebsiella pneumoniae* carbapenemase-producing 609803** | | | | | | | | | | |
| Inhibition at acid pH, SD (%) | 100 ± 2.72 | 100 ±0.67 | 98.54 ± 3.76 | 100 ± 3.12 | 99.65 ± 1.81 | 100 ± 0.28 | 97.41 ± 3.39 | 100 ± 1.46 | 100 ± 0.54 | 100 ± 1.33 |
| Inhibition at neutral pH, SD (%) | 6.75 ± 2.94 | 18.57 ± 3.44 | 17.37 ± 4.47 | 21.55 ± 3.47 | 18.90 ± 5.17 | 31.29 ± 5.06 | 17.79 ± 2.71 | 12.70 ± 3.02 | 13.72 ± 3.38 | 17.21 ± 5.27 |
| Wilcoxon test *p*-value | 0.0006306361 | 0.0004038323 | 4.113534E-05 | 0.0006306361 | 0.0004066393 | 0.0003818697 | 0.0004038323 | 0.0004066393 | 0.0006209699 | 0.0003954938 |
| ***Pseudomonas aeruginosa* ATCC 27853** | | | | | | | | | | |
| Inhibition at acid pH, SD (%) | 93.99 ± 2.07 | 100 ± 1.92 | 97.09 ± 4.71 | 95.37 ± 1.64 | 98.07 ± 3.04 | 100 ± 2.02 | 94.74 ± 2.13 | 100 ± 1.10 | 97.28 ± 2.70 | 99.91 ± 2.24 |
| Inhibition at neutral pH, SD (%) | -0.17 ± 2.99 | -1.61 ± 3.20 | 11.90 ± 3.37 | -2.03 ± 4.41 | 7.00 ± 4.94 | -7.83 ± 5.02 | -0.19 ± 4.51 | -3.89 ± 4.19 | -3.76 ± 4.33 | -0.12 ± 3.44 |
| Wilcoxon test *p*-value | 4.113534E-05 | 0.0004094601 | 4.113534E-05 | 4.113534E-05 | 4.113534E-05 | 0.0004094601 | 4.113534E-05 | 0.0003954938 | 4.113534E-05 | 0.0004094601 |
| ***Pseudomonas aeruginosa* P28** | | | | | | | | | | |
| Inhibition at acid pH, SD (%) | 99.55 ± 4.77 | 100 ± 2.28 | 97.08 ± 3.50 | 98.10 ± 2.83 | 97.57 ± 4.87 | 100 ± 3.31 | 91.66 ± 2.88 | 93.72 ± 1.49 | 98.84 ± 2.45 | 99.04 ± 4.05 |
| Inhibition at neutral pH, SD (%) | -0.59 ± 5.49 | -0.03 ± 5.14 | -0.83 ± 4.12 | -1.94 ± 5.38 | -2.13 ± 4.33 | -0.49 ± 4.41 | -6.61 ± 1.71 | -6.77 ± 2.54 | -5.90 ± 3.26 | -1.23 ± 3.43 |
| Wilcoxon test *p*-value | 4.113534E-05 | 0.0004066393 | 0.0004094601 | 8.227067E-05 | 0.0004094601 | 4.113534E-05 | 0.000401039 | 0.0006257901 | 0.0004038323 | 0.0004066393 |
| ***Staphylococcus aureus* ATCC 25923** | | | | | | | | | | |
| Inhibition at acid pH, SD (%) | 98.30 ± 2.33 | 100 ± 1.02 | 98.37 ± 1.47 | 97.89 ± 2.60 | 100 ± 5.25 | 100 ± 2.19 | 94.20 ± 3.28 | 100 ± 4.44 | 100 ± 2.53 | 98.74 ± 1.60 |
| Inhibition at neutral pH, SD (%) | 2.18 ± 4.36 | 20.26 ± 3.82 | 35.44 ± 4.74 | 18.04 ± 5.09 | 31.84 ± 4.45 | 36.47 ± 4.83 | 2.48 ± 5.43 | 20.72 ± 4.69 | 14.49 ± 4.26 | 7.96 ± 5.23 |
| Wilcoxon test *p*-value | 0.0001748252 | 0.0006257901 | 0.0004038323 | 0.0004094601 | 4.113534E-05 | 8.227067E-05 | 8.227067E-05 | 0.0001554002 | 0.000401039 | 8.227067E-05 |
| **Methicillin-resistant *Staphylococcus aureus* 333** | | | | | | | | | | |
| Inhibition at acid pH, SD (%) | 92.14 ± 4.43 | 96.06 ± 4.55 | 99.87 ± 1.23 | 92.17 ± 1.28 | 99.62 ± 2.44 | 100 ± 1.74 | 91.85 ±1.36 | 98.56 ± 3.25 | 99.85 ± 1.33 | 100 ± 4.67 |
| Inhibition at neutral pH, SD (%) | -1.24 ± 4.69 | 8.66 ± 4.99 | 16.44 ± 2.80 | 12.06 ± 3.63 | 14.97 ± 4.77 | 12.70 ± 4.35 | 2.09 ± 3.41 | -1.95 ± 2.88 | -0.71 ± 3.64 | -4.98 ± 4.62 |
| Wilcoxon test *p*-value | 0.0004094601 | 8.227067E-05 | 0.0004094601 | 0.0001554002 | 8.227067E-05 | 0.0004094601 | 0.0006306361 | 4.113534E-05 | 0.0004066393 | 0.0004066393 |
| ***Enterococcus faecalis* ATCC 29212** | | | | | | | | | | |
| Inhibition at acid pH, SD (%) | 100 ± 2.43 | 100 ± 2.87 | 100 ± 4.26 | 100 ±3.67 | 100 ± 4.47 | 100 ± 2.84 | 97.86 ± 3.37 | 100 ± 3.27 | 100 ± 1.73 | 100 ± 3.74 |
| Inhibition at neutral pH, SD (%) | 10.19 ± 4.61 | -1.88 ± 2.44 | 7.73 ± 3.08 | 8.05 ±3.81 | 11.86 ± 3.92 | -4.01 ± 168 | -1.74 ± 4.68 | 8.98 ± 4.96 | 14.08 ± 4.20 | -2.30 ± 5.51 |
| Wilcoxon test *p*-value | 4.113534E-05 | 0.000401039 | 8.227067E-05 | 4.113534E-05 | 0.0004066393 | 0.0004094601 | 0.0004094601 | 0.0004066393 | 0.0004066393 | 0.0004094601 |
| **Vancomycin-resistant *Enterococcus faecalis* INSPI 032** | | | | | | | | | | |
| Inhibition at acid pH, SD (%) | 95.16 ± 3.31 | 100 ±3.21 | 100 ± 3.40 | 97.02 ± 4.59 | 94.28 ± 2.32 | 100 ± 3.30 | 99.16 ± 3.78 | 99.80 ± 3.52 | 100 ± 3.01 | 100 ± 4.77 |
| Inhibition at neutral pH, SD (%) | -2.36 ± 4.72 | -3.90 ± 4.77 | 39.02 ± 4.58 | -5.29 ± 3.92 | 28.17 ± 4.27 | -5.47 ± 4.80 | 35.41 ± 4.59 | -0.05 ± 3.55 | -3.58 ± 5.21 | -0.97 ± 5.16 |
| Wilcoxon test *p*-value | 0.001024163 | 0.0004066393 | 4.113534E-05 | 4.113534E-05 | 0.0004094601 | 8.227067E-05 | 0.0006257901 | 0.0004094601 | 0.0004066393 | 0.000401039 |
| ***Enterococcus faecium* ATCC 27270** | | | | | | | | | | |
| Inhibition at acid pH, SD (%) | 99.26 ± 3.45 | 97.16 ± 4.36 | 98.36 ± 3.34 | 97.38 ± 4.34 | 97.30 ± 1.57 | 99.82 ± 1.78 | 98.23 ± 3.15 | 100 ± 1.22 | 97.06 ± 4.37 | 99.94 ± 2.23 |
| Inhibition at neutral pH, SD (%) | -4.63 ± 3.17 | -4.93 ± 3.78 | -4.60 ± 3.38 | -4.53 ± 2.81 | 0.06 ± 2.99 | -4.95 ± 3.74 | -4.64 ± 3.17 | -4.98 ± 3.21 | -3.81 ± 3.05 | 0.45 ± 1.32 |
| Wilcoxon test *p*-value | 4.113534E-05 | 0.0004094601 | 0.0006306361 | 8.227067E-05 | 0.0004094601 | 0.0004066393 | 4.113534E-05 | 4.113534E-05 | 4.113534E-05 | 0.0004094601 |
| ***Candida albicans* ATCC 10231** | | | | | | | | | | |
| Inhibition at acid pH, SD (%) | 99.23 ± 1.72 | 100 ± 1.02 | 100 ± 4.77 | 99.05 ± 2.14 | 100 ± 2.31 | 100 ± 1.36 | 85.79 ± 3.08 | 99.83 ± 2.29 | 98.91 ± 3.94 | 100 ± 1.28 |
| Inhibition at neutral pH, SD (%) | -3.29 ± 4.32 | -3.38 ± 3.21 | -4.19 ± 1.85 | -3.05 ± 3.79 | 10.60 ± 4.38 | -3.05 ± 2.43 | -3.30 ± 3.61 | -3.85 ± 3.69 | -3.52 ± 4.35 | -3.08 ± 2.24 |
| Wilcoxon test *p*-value | 4.113534E-05 | 0.000401039 | 4.113534E-05 | 0.0004038323 | 0.0006209699 | 0.0003982596 | 4.113534E-05 | 4.113534E-05 | 0.0004094601 | 0.0006257901 |
| ***Candida tropicalis* WT** | | | | | | | | | | |
| Inhibition at acid pH, SD (%) | 81.78 ± 1.26 | 99.23 ± 4.51 | 92.70 ± 4.94 | 85.21 ± 4.77 | 86.84 ± 3.66 | 87.80 ± 3.21 | 87.82 ± 2.27 | 84.73 ± 2.60 | 87.00 ± 3.22 | 84.59 ± 2.77 |
| Inhibition at neutral pH, SD (%) | -7.04 ± 4.52 | -7.68 ± 3.72 | -6.06 ± 3.61 | -7.95 ± 3.09 | -6.09 ± 4.16 | -7.36 ± 4.30 | -7.93 ± 2.29 | -5.37 ± 5.05 | -5.92 ± 2.81 | -6.48 ± 3.35 |
| Wilcoxon test *p*-value | 0.0003845673 | 4.113534E-05 | 4.113534E-05 | 8.227067E-05 | 0.0004094601 | 0.0004066393 | 0.0004066393 | 8.227067E-05 | 0.0004094601 | 0.0001748252 |
| ***Candida tropicalis* INSPI 024** | | | | | | | | | | |
| Inhibition at acid pH, SD (%) | 85.12 ± 3.77 | 82.49 ± 1.57 | 89.70 ± 4.63 | 81.14 ± 2.6 | 83.33 ± 4.13 | 87.34 ± 3.70 | 82.51 ± 2.42 | 82.46 ± 3.02 | 84.11 ± 2.49 | 92.02 ± 3.12 |
| Inhibition at neutral pH, SD (%) | -1.90 ± 4.22 | -1.79 ± 3.46 | -2.64 ± 3.48 | -3.13 ± 4.02 | -3.02 ± 4.43 | -4.74 ± 4.49 | -3.21 ± 4.41 | -3.61 ± 3.53 | -2.96 ± 4.70 | -4.81 ± 4.08 |
| Wilcoxon test *p*-value | 0.0004094601 | 0.0003954938 | 0.0004094601 | 0.0004066393 | 0.0004094601 | 4.113534E-05 | 0.0004094601 | 0.0006306361 | 0.0004094601 | 0.0006306361 |

The mean percentage inhibition and standard deviation (SD) were calculated using the mean values of the total number of assays (triplicate on three independent experiments). Negative inhibition percentages indicate that there was greater growth than the control without treatment. The Wilcoxon test was selected to evaluate the statistical differences between percentage inhibition at acid pH vs percentage inhibition at neutral pH of different CFS.
