# Supplementary material for: Cell-Free but potent: Antimicrobial potential of Lactobacillus supernatants against multidrug-resistant pathogens
Source: PLoS One. 2026 Aug 25;21(8):e0356730. doi: 10.1371/journal.pone.0356730 (PMC13505906; doi:10.1371/journal.pone.0356730)
Supplement: Supplementary file 12 [file pone.0356730.s012.docx]

**Supplementary Table S12.** Cell viability response to CFS as assessed by MTT assay.

|  | **HEP G2 cell line** | | | **RAW 264.7 cell line** | | |
| --- | --- | --- | --- | --- | --- | --- |
|  | **Cell viability ± SD (%)** | **Wilcoxon test *p*-value** | | **Cell viability ± SD (%)** | **Wilcoxon test *p*-value** | |
|  |  | **With CFS vs Control without CFS** | **With CFS vs Eluent control** |  | **With CFS vs Control without CFS** | **With CFS vs Eluent control** |
| **Effective CFS** | | | | | | |
| *L. plantarum* IM-USFQ | 97.18 ± 3.18 | 0.946434 | 0.2175534 | 40.44 ± 5.19 | 0.0005429155 | 8.227067e-05 |
| *L. plantarum* NCCB 46043^T^ | 83.03 ± 6.12 | 0.002132086 | 0.0375239 | 48.89 ± 4.12 | 0.001917013 | 0.0006660007 |
| *L. acidophilus* IM-USFQ | 93.69 ± 5.73 | 0.2094824 | 0.897082 | 44.98 ± 5.94 | 0.001191504 | 0.0003108003 |
| *L. acidophilus* ATCC 4356^T^ | 98.21 ± 4.72 | 0.301389 | 0.06547173 | 35.75 ± 5.70 | 0.001191504 | 0.0003108003 |
| *L. gasseri* IM-USFQ | 94.72 ± 5.10 | 0.382439 | 0.5138875 | 47.82 ± 4.39 | 0.001191504 | 0.0003108003 |
| *L. gasseri* V254 | 90.46 ± 5.69 | 0.01290634 | 0.6018749 | 38.94 ± 6.87 | 0.001191504 | 0.0003108003 |
| *L. gasseri* V140B | 95.22 ± 5.18 | 0.4372186 | 0.5177988 | 59.95 ± 5.60 | 0.001917013 | 0.007992008 |
| *L. crispatus* V118C | 94.05 ± 6.31 | 0.3096559 | 0.897082 | 44.69 ± 6.84 | 0.001917013 | 0.0006660007 |
| *L. iners* ATCC 55195 | 95.47 ± 4.45 | 0.382439 | 0.3457176 | 48.96 ± 4.20 | 0.001191504 | 0.0003108003 |
| *L. reuteri* NCFB 2656^T^ | 91.31 ± 4.92 | 0.02923924 | 0.6999043 | 49.80 ± 4.46 | 0.001191504 | 0.0003108003 |
| **Ineffective CFS** | | | | | | |
| *L. gasseri* V189D | 80.91 ± 5.94 | 0.0008598969 | 0.004505689 | 63.82 ± 5.50 | 0.001917013 | 0.05927406 |
| *L. gasseri* V193B | 96.79 ± 5.42 | 0.8506523 | 0.230776 | 45.59 ± 6.54 | 0.001191504 | 0.0003108003 |
| *L. gasseri* V239B | 92.65 ± 5.03 | 0.08333178 | 0.8698586 | 66.81 ± 4.58 | 0.001191504 | 0.231857 |
| *L. crispatus* V342B | 99.22 ± 2.07 | 0.190518 | 0.02338377 | 50.55 ± 3.49 | 0.0007781335 | 0.0009309723 |
| *L. fermentum* ATCC 11739^T^ | 92.30 ± 4.58 | 0.0287451 | 0.9536082 | 53.69 ± 4.75 | 0.001191504 | 0.0006216006 |
| **Controls** | | | | | | |
| Control without CFS | 97.27 ± 3.57 | | | 97.94 ± 3.21 | | |
| Eluent control | 92.59 ± 5.51 | | | 70.49 ± 5.31 | | |
| *p*-value (control without CFS vs Eluent control) | 0.1062896 | | | 0.0007853 | | |

The mean percentage of cell viability and standard deviation (SD) were calculated using the mean values of the total number of assays (triplicate on three independent experiments). The Wilcoxon test was selected to evaluate the statistical differences between percentage without treatment vs percentage with treatment (different CFS and controls).
